# Loss of sex-determining region Y-box 2 (Sox2) captures embryonic stem cells in a primed pluripotent state

**DOI:** 10.1016/j.jbc.2025.108501

**Published:** 2025-04-09

**Authors:** Min Qi, Bowen Wang, Huaqi Liao, Yuzhuo Xu, Lixia Dong, Lijun Xu, Yin Xia, Xiaochun Jiang, Shizhang Ling, Jinzhong Qin

**Affiliations:** 1 State Key Laboratory of Pharmaceutical Biotechnology and MOE Key Laboratory of Model Animals for Disease Study, Model Animal Research Center, Medical School of Nanjing University, Nanjing, China; 2 School of Biomedical Sciences, The Chinese University of Hong Kong, Hong Kong, China; 3 The Translational Research Institute for Neurological Disorders, Department of Neurosurgery, The First Affiliated Hospital (Yijishan Hospital) of Wannan Medical College, Wannan Medical College, Wuhu, China; 4 Jiangsu Key Laboratory of Molecular Medicine, Medical School of Nanjing University, Nanjing, China

**Keywords:** Sox2, embryonic stem cells, epiblast stem cells, Sox3, transition, pluripotency, redundancy

## Abstract

Two main pluripotent cell lines can be established from the preimplantation and postimplantation mouse embryo as naïve embryonic stem cells (ESCs) and primed epiblast stem cells (EpiSCs), respectively. Although the two pluripotent states are interconvertible, the molecular mechanism controlling the transition between naïve and primed pluripotency remains to be fully elucidated. Here, by performing a CRISPR-based loss-of-function screen in ESCs, we identify Sox2 involved in the repression of lineage-specification marker brachyury (T). Upon Sox2 ablation in ESCs, two populations of cells mutually exclusive for CDX2 (trophectoderm marker) and T expression can be observed. T-positive cells display features resembling the salient characteristics of EpiSCs including molecular and functional properties. By using genetic ablation approach, we show that acquisition and maintenance of primed pluripotency in Sox2 null T-positive cells heavily depend on fibroblast growth factor (Fgf) and Nodal, which is produced in an autocrine manner in these cells. We further demonstrate that Sox3 compensates for the absence of Sox2 in maintaining the primed state of Sox2-null pluripotent cells. Establishment of Sox2-deficient pluripotent cells will enable the elucidation of the mechanisms controlling the transition of cells between different states of pluripotency.

At the time of implantation, the blastocyst (the early mammalian embryo) consists of three distinct cell lineages: the epiblast (EPI), the primitive endoderm (PrE, also called the hypoblast), and the trophectoderm (TE) (1, 2). These three founding lineages are thought to arise through two consecutive rounds of binary cell fate decisions. During the first cell fate decision in mouse embryo (from 8-cell to 32-cell stage), the extra-embryonic TE lineage (from which the placenta ultimately develops) is segregated from inner cell mass (ICM). In the second cell fate decision occurring at the blastocyst stage in the mouse, the pluripotent ICM differentiates into the extraembryonic PrE (a predecessor of the yolk sac), and the pluripotent EPI, which eventually gives rise to the embryo proper (2). Pluripotency is defined as the ability of a cell to give rise to cells of the three germ layers, namely, ectoderm, endoderm, and mesoderm, which together give rise to a mature organism. Pluripotency is a transient property of embryonic cells within the early embryo. *In vitro*, however, different pluripotent stem cell lines representing distinct stages of embryogenesis can be derived from the early embryo, and maintained indefinitely in an artificially induced self-renewal state under strictly defined conditions.

Mouse embryonic stem cells (ESCs), routinely derived from the inner cell mass (ICM) of developing blastocyst, can be propagated indefinitely *in vitro* in a naive ground state of pluripotency, as they retain the capacity to contribute to all somatic and germline cell types when injected in the host blastocyst (3, 4). The undifferentiated state of ESCs depends on an interconnected regulatory circuit formed by Oct4 (encoded by Pou5f1)/Sox2/Nanog and LIF/STAT3-dependent signaling pathways, which safeguard ESC self-renewal and prevent Fgf-mediated lineage specification. EpiSCs, on the other hand, are isolated from the postimplantation mouse embryo just after implantation but prior to gastrulation and exhibit an alternative pluripotency configuration, referred to a primed pluripotency (5). Although EpiSCs can form teratomas containing tissue derivatives of all three embryonic germ layers, they have negligible capacity to contribute to chimeras and are unable to contribute to the germline (5, 6, 7). Unlike ESCs, EpiSCs do not utilize the LIF/Stat3 pathway and instead rely on Fgf and Activin (6, 7). Grown as large flat colonies, they are morphologically distinct from domed mouse ESC colonies. Unlike ESCs, EpiSCs are sensitive to single-cell dissociation and are usually passaged as clusters of cells rather than dispersed. EpiSCs exhibit epiblast markers such as Fgf5, Cer1 and T, which are low or absent in ESCs (5, 6, 8), and show inactivation of the X chromosome in female cells. Intriguingly, although derived from the ICM, the human ESCs (hESCs) resemble mouse EpiSCs in terms of their colony morphology, X chromosome inactivation status, as well as signaling requirements for maintaining pluripotency (5).

The pluripotent state of ESCs is maintained through a coordinated action of a core set of transcription factors that include the well-studied Oct4, Sox2, Nanog, and Klf4, together with multiple signaling pathways in response to environmental cues (9). In comparison, less is known about the molecular mechanism governing the exit from the naïve pluripotent state and the transcriptional circuits that enable EpiSCs to maintain primed pluripotency. ChIP analysis of Oct4 targets in EpiSCs showed little overlap with the targets in mouse ESCs (6). Accordingly, the number of proteins in complex with Oct4, Sox2, and Nanog in mouse ESCs are significantly decreased or not detected in EpiSCs including Nr0b1 (Dax1), Esrrb, and Klf4(6). Recently, Otx2 has also been emphasized as a key transcription factor driving naïve to primed transition, at least in part, through cooperative interactions with Oct4 (10, 11, 12). Additionally, Zic2/3 has been proposed to function as transcriptional determinants of EpiSCs (13, 14). Here we show that the ablation of Sox2 in ESCs results in two major mutually exclusive subpopulations characterized by the expression of trophectoderm (CDX2) and epiblast (T) markers, respectively. Detailed analysis of the T-positive cells revealed that they have entered a primed pluripotent state with unique features that resemble EpiSCs and with distinct morphological, signaling, and functional properties from ESCs. We further show that the loss of both Sox2 and Sox3 results in the complete abrogation of self-renewal and pluripotency of ESCs and demonstrate that the elevated Sox3 expression functionally compensates for the loss of Sox2 in Sox2 null cells reminiscent of EpiSCs.

## Results

### A loss-of-function screen identifies Sox2 as a candidate gene in coordinating the transition from naïve to primed pluripotency

**Figure 1:**
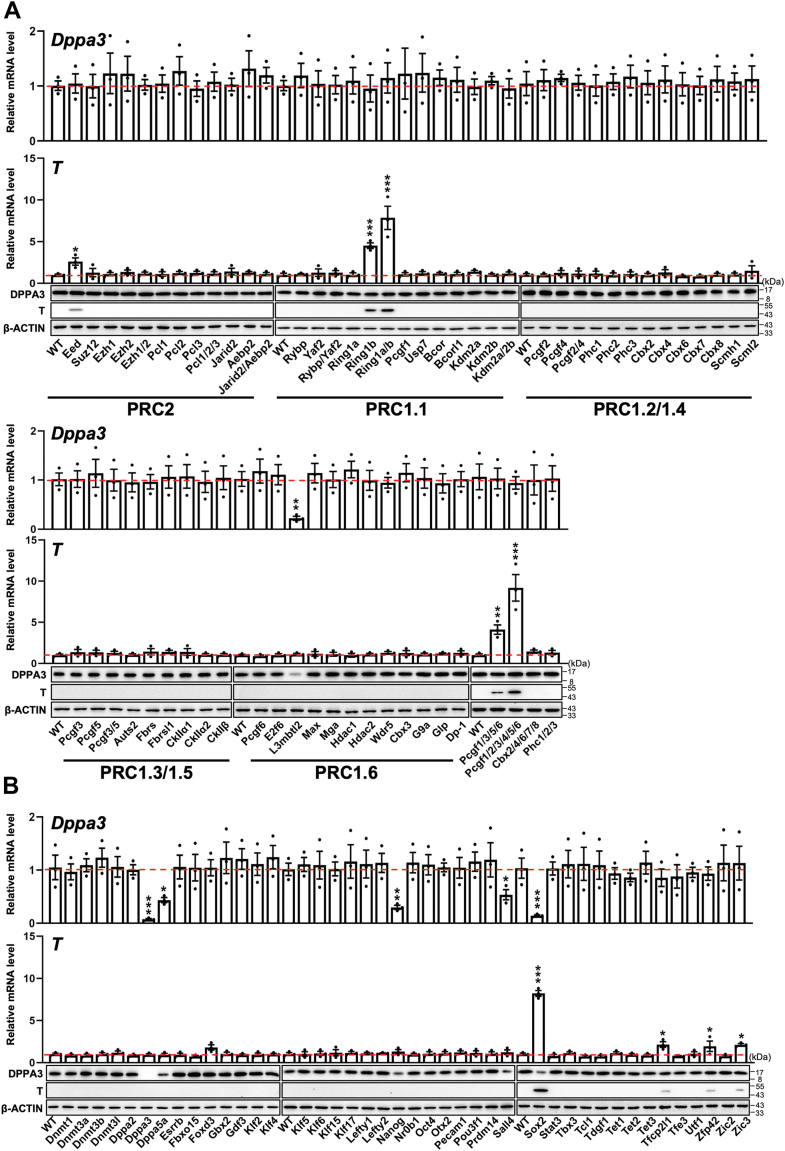
**Effect of ablation of PcG genes or pluripotency factors on the expression of Dppa3 and T in ESCs**. *A* and *B*, the effect of deletion of PcG genes (*A*) or CRISPR-mediated knockout of pluripotency factors on the expression of Dppa3 and T in ESCs determined by RT-qPCR (*top*) and Western blot (*bottom*). The mean value of three replicates was normalized using β-actin as internal controls and the expression level in wild-type (WT) ESCs was arbitrarily set to 1.0. β-Actin served as a loading control. Data were obtained from three independent experiments and expressed as the mean ± SD. (∗*p* < 0.05, ∗∗*p* < 0.01, ∗∗∗*p* < 0.001).

Given the observation that EpiSCs acquire histone H3 Lys27 trimethylation (H3K27me3) marks over developmental genes (5, 15), we explored the role of Polycomb group (PcG) proteins, which deposit H3K27me3, in protecting ESCs from a primed state of pluripotency. To this end, we first screened for members in the PcG family (16, 17), whose deficiency in ESCs led to increased levels of EpiSC marker T (5, 6, 8) and attenuated expression of Dppa3 (also known as Stella or Pgc7), a naive pluripotency marker (5), by using available ESC lines harboring loss of function mutations in PcG (16, 17). We found that although lack of Eed, Ring1b, Ring1a/b, Pcgf1/3/5/6, or Pcgf1-6 in ESCs resulted in increased expression of T at both mRNA and protein levels, the expression of Dppa3 genes remained unchanged (Fig. 1*A*). Additionally, despite the reduced levels of Dppa3, L3mbtl2 elimination did not affect T expression. These results suggest that polycomb is not a central mechanism for the transition from naïve to primed pluripotency.

We then conducted a CRISPR/Cas9-based screen in C57BL/6-derived ESCs against the 42 selected transcriptional and epigenetic factors previously evaluated in the context of naïve and primed pluripotency maintenance (5, 18) (Fig. S1). The inactivation of five out of 42 selected genes resulted in a significantly decreased expression of Dppa3 gene, including Dppa5a, Nanog, Sall4, and Sox2, supporting the vital role of these candidates in pluripotency maintenance (Fig. 1*B*). Among these candidate genes, only the inactivation of the Sox2 gene also resulted in a significant induction of T. We then focused on Sox2, which has not been implicated in naïve to primed transition.

### Sox2 and Sox3 play redundant but collectively essential roles in the maintenance of pluripotency

**Figure 2:**
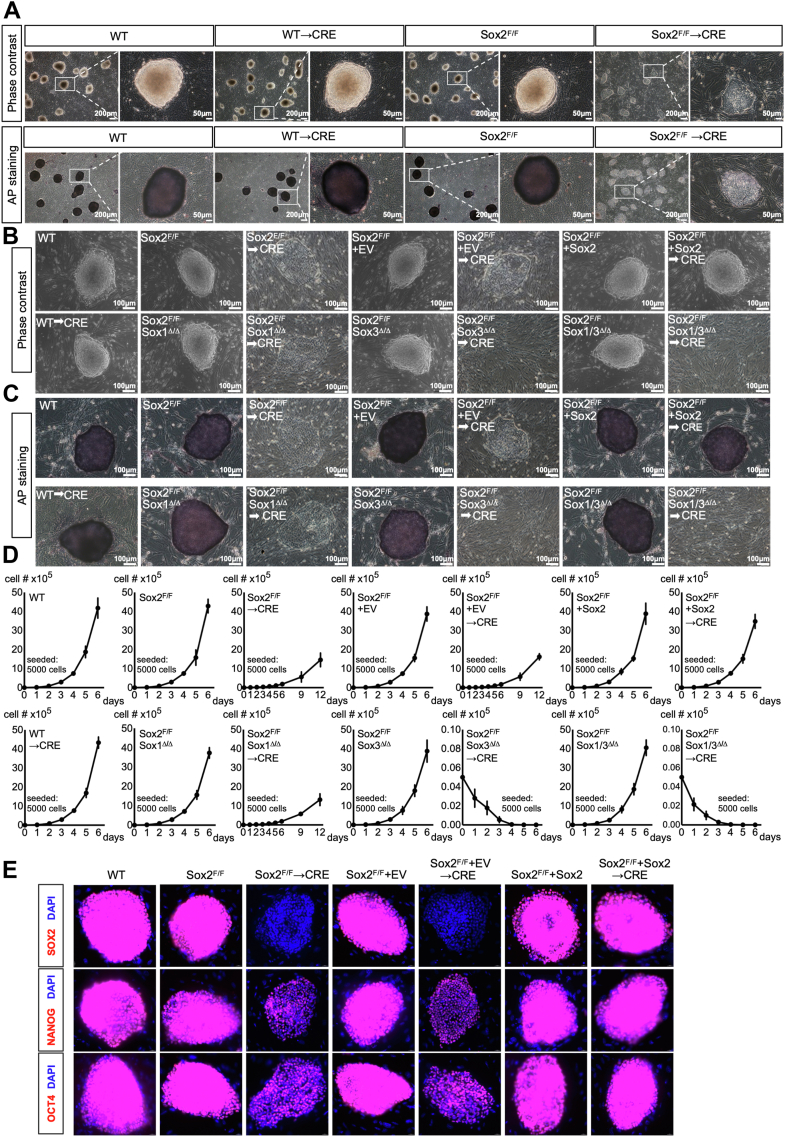
**The phenotypic defects observed in ESCs deficient in Soxb1 family genes**. *A*, representative phase contrast pictures (*top*) and AP staining images (*bottom*) of ESC colonies of indicated genotypes on MEF feeder layer. A higher magnification image of the boxed area is shown on the *right*. *B*, phase-contrast images of ESC colonies grown on a feeder layer of MEFs. *C*, representative images of AP staining of ESC colonies of indicated genotypes cultured on a feeder layer of MEFs. The scale bar is shown in the image of the *bottomright* corner. *D*, the representative growth curve of ESCs of indicated genotypes. Data represent the mean ± SD of three independent experiments. *E*, Immunofluorescence analysis for OCT4, NANOG, SOX2 (*red*), or DAPI (*blue*) in ESC colonies of indicated genotypes. The images were captured using confocal microscopy with a 63x objective lens. All images were shown merged with the *blue* and *red* fluorescent channels. Shown is representative of three independent experiments, each with two different clones analyzed.

The mouse genome harbors 20 Sox family members, which are categorized into eight groups. Sox2 belongs to group B1 (SoxB1), which has two other members, Sox1 and Sox3 (19)). To circumvent the cell lethality resulting from complete disruption of the Sox2 gene (20), (we, therefore, established a C57BL/6-derived ESC line harboring Sox2 conditional null alleles) we established Sox2 conditional floxed ESC line (Sox2^F/F^) by flanking the single exon of the Sox2 gene on both alleles with loxP sites, based on Cre-loxP system (Fig. S2*A*). To elucidate potential functional redundancy among the SoxB1 family members, we engineered Sox1^Δ/Δ^ or/and Sox3^Δ/Δ^ ESCs with conditional Sox2^F/F^ (Sox1^Δ/Δ^;Sox2^F/F^, Sox3^Δ/Δ^;Sox2^F/F^ and Sox1/3^Δ/Δ^;Sox2^F/F^) (Fig. S2, *B* and *C*). For comparison, we also generated conditional Oct4-and Nanog-null ESCs using a similar approach (Fig. S2, *D* and *E*). Sox2^F/F^ ESCs retained high expression levels of the pluripotency markers such as Sox2, Oct4 or Nanog, and formed compact and dome-shaped colonies with defined borders when cultured on a feeder layer from mitotically inactivated mouse embryonic fibroblast (MEF) cells, and stained strongly positive for Alkaline phosphatase (AP) activity (Figs. 2, *A*–*E* and S3*A*, and see below for more details). Sox2 was completely removed 48h after infection by lenti-Cre virus, as evident from Western blot analysis (Fig. S2*A*). Therefore, the cells were used for assay after 48 h of Cre infection throughout this study, unless otherwise stated. The deletion of the Sox2 gene in ESCs (Sox2^Δ/Δ^) resulted in an accumulation of cells in the G0/G1 phase, a reduction of S phase cells, and a concomitant increase in cells undergoing apoptosis (Figs. 2*D*, S3, *B* and *C*). Upon plating of Sox2^Δ/Δ^ cells onto a feeder layer, they underwent a process of spontaneous differentiation, as evidenced by a flattened and spread cell morphology and by loss of AP staining (Fig. 2, *A*–*C*). The phenotypes observed in Sox2^Δ/Δ^ cells were specific to Sox2 gene deficiency since they were rescued by the ectopic expression of wild-type Sox2 using lentivirus-mediated transduction. In contrast, the single or double mutants deficient in the Sox1 or/and Sox3 genes retained a typical undifferentiated state in terms of colony morphology along with a high expression level of AP. The established Sox1^Δ/Δ^;Sox2^F/F^, Sox3^Δ/Δ^;Sox2^F/F^ and Sox1/3^Δ/Δ^;Sox2^F/F^ formed tightly compact colonies, stained highly positive for AP (Fig. 2, *A*–*C*). However, Cre-mediated ablation of Sox2 from Sox3^Δ/Δ^; Sox2^F/F^ and Sox1/3^Δ/Δ^; Sox2^F/F^, but not Sox1^Δ/Δ^; Sox2^F/F^, ESCs led to a severe decrease in their rate of proliferation relative to control Cre-infected cells, with increase of G0/G1 phase arrest, alongside an increase in apoptosis (Fig. S3, *B* and *C*). Upon plating these cells onto a feeder layer, Sox2/3^Δ/Δ^ and Sox1/2/3^Δ/Δ^ underwent immediate differentiation and did not form any colony after 4 weeks of culture, whereas Sox1/3^Δ/Δ^ displayed a behavior similar to that of Sox2^F/F^ (Fig. 2, *B* and *C*). These results thus uncovered a degree of functional redundancy between Sox2 and Sox3, but not Sox1, in preserving ESC pluripotent identity.

**Figure 3:**
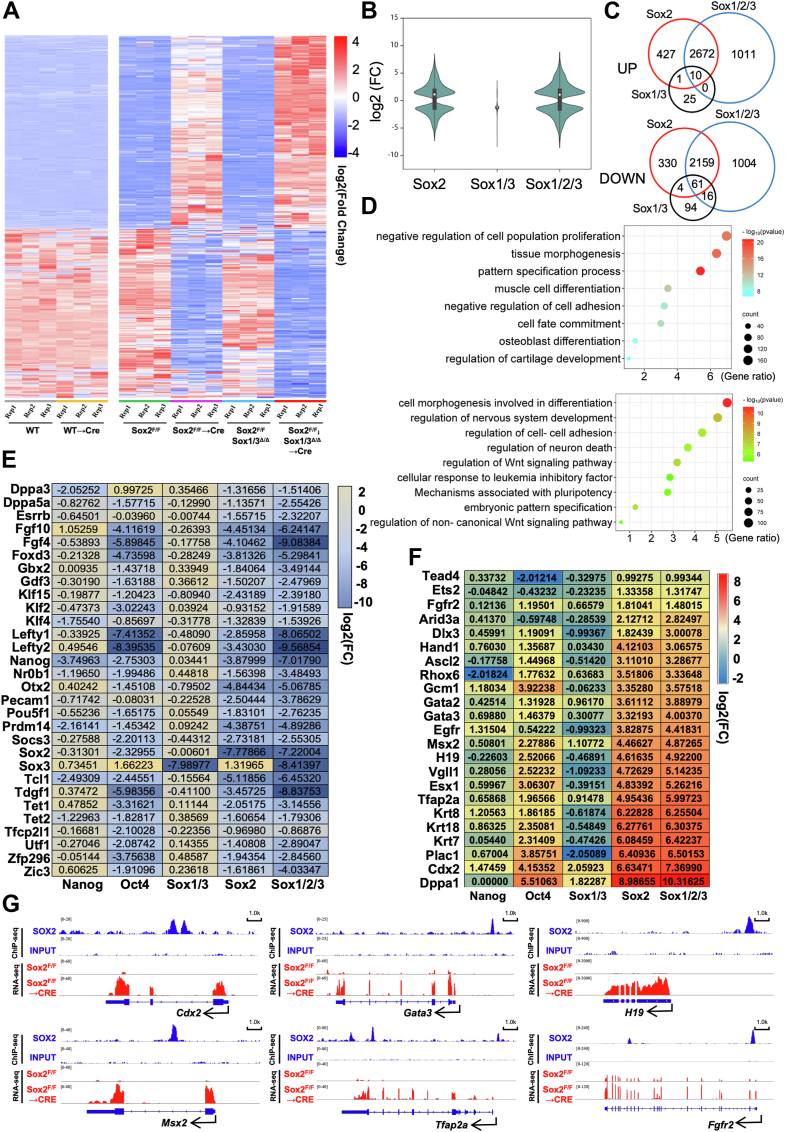
**Sox2 and Sox3 exert redundant effects on the control of pluripotency**. *A*, heatmap depicting fold changes in gene expression in ESCs of indicated genotypes. False discovery rate (FDR) < 0.05. Transcripts of significantly up- and down-regulated genes are highlighted *red* and *blue*, respectively. *B*, a violin plot comparing log2-fold changes of genes in ESCs deficient for Sox2, Sox1/3, and Sox1/2/3. *C*, Venn diagram showing overlap of upregulated (*top*) or downregulated (*bottom*) genes between Sox2^Δ/Δ^, Sox1/3^Δ/Δ^, and Sox1-3^Δ/Δ^ ESCs. *D*, GO analysis of genes up-regulated (*top*) and down-regulated (*bottom*) in Sox2^F/F^ cells transfected with Cre for 72h. *E* and *F*, Heatmaps showing the expression levels of genes involved in (E) pluripotency maintenance and (F) trophectoderm lineage specification in ESCs of indicated genotypes. *G*, genomic snapshots of genes associated with TE development, showing Sox2 ChIP-seq and RNA-seq in Sox2^F/F^ cells (before and after Cre transfection). Published ChIP-seq data for Sox2 were obtained from the NCBI GEO database (GEO: GSE81168).

To gain insight into the potential mechanisms underlying the phenotypic changes, we carried out RNA-seq analysis in Sox1/3^Δ/Δ^ and Sox2^F/F^ or Sox1/3^Δ/Δ^; Sox2^F/F^ before and after Cre infection (Fig. 3, *A* and *B*, S4*A* and Table S1-S5). Only 175 genes were down-regulated in Sox1/3^Δ/Δ^ compared with 2554 genes in Sox2^Δ/Δ^ and 3240 genes in Sox1/2/3^Δ/Δ^ ESCs (Fig. 3*C*). Genes downregulated in Sox2^Δ/Δ^, but not Sox1/3^Δ/Δ^, largely overlapped with the downregulated genes in Sox1/2/3^Δ/Δ^ ESCs. As a control, Cre expression had no effect on gene expression profile in Wildtype ESCs (Figs. 3*A* and S4*A*). Gene ontology (GO) analysis revealed that genes down-regulated in Sox2-deficient ESCs were strongly associated with the regulation of the Wnt signaling pathway, mechanisms associated with pluripotency, as well as cell morphogenesis involved in differentiation (Fig. 3*D*). Furthermore, we found that a cohort of pluripotency-associated genes previously described to be direct targets of Oct4 was also down-regulated in ESCs lacking Sox2, but not Nanog (Fig. 3*E* and Table S1-S3), consistent with the notion that Sox2 and Oct4 form a heterodimer to cooperatively activate pluripotency-related genes, including themselves (21). Importantly, consistent with the observed phenotype, the removal of Sox2 in addition to Sox1/3 had a strong additive effect on the expression levels of pluripotency-associated genes (Fig. 3*E*). Focusing on significantly upregulated genes, we identified 36 genes in Sox1/3^Δ/Δ^, 3110 genes in the Sox2^Δ/Δ,^ and 3693 genes in Sox1/2/3^Δ/Δ^ *versus* control ESCs (Fig. 3*C*). The majority of genes up-regulated in Sox2^Δ/Δ^, but not Sox1/3^Δ/Δ^, cells were also up-regulated in Sox1/2/3^Δ/Δ^ cells (Fig. 3*C*). Functional annotation of genes up-regulated in Sox2^Δ/Δ^ ESCs showed high enrichment of GO terms related to pattern specification process, cell fate commitment, and tissue morphogenesis (Fig. 3*D*). Among the up-regulated transcripts, we found that panels of trophectoderm-specific genes previously described to be controlled by Oct4 were also highly derepressed in ESCs lacking Sox2 (22), but not Nanog, further supporting a close association between Sox2 ablation and the acquisition of TE identity (23) (Fig. 3*F*). Upon examination of publicly available Sox2-bound targets defined by ChIP-seq in ESCs, multiple TE-associated genes such as Cdx2, Gata3, Msx2, and Tfap2a were identified as direct targets of Sox2 (Fig. 3*G*). Conversely, expression levels of ectodermal (Pax6, Gfap, Neurod1, and Olig1) or mesendoderm (Flk1, Eomes, Hnf1b, and Mixl1) markers either not enhanced or only weakly increased after Cre infection (Table S1). Notably, Sox2^Δ/Δ^ ESCs expressed low (moderate) levels of epiblast markers, such as Cer1, Eomes, Fgf5, Sox17, and T, when compared to ESCs. Together, our results show that Sox2 shields naïve pluripotent cells from trophectoderm differentiation and that the Sox2 function in pluripotency is redundantly supported by its paralog Sox3.

### Sox2-deficient stable cell lines exist in a primed state of pluripotency

**Figure 4:**
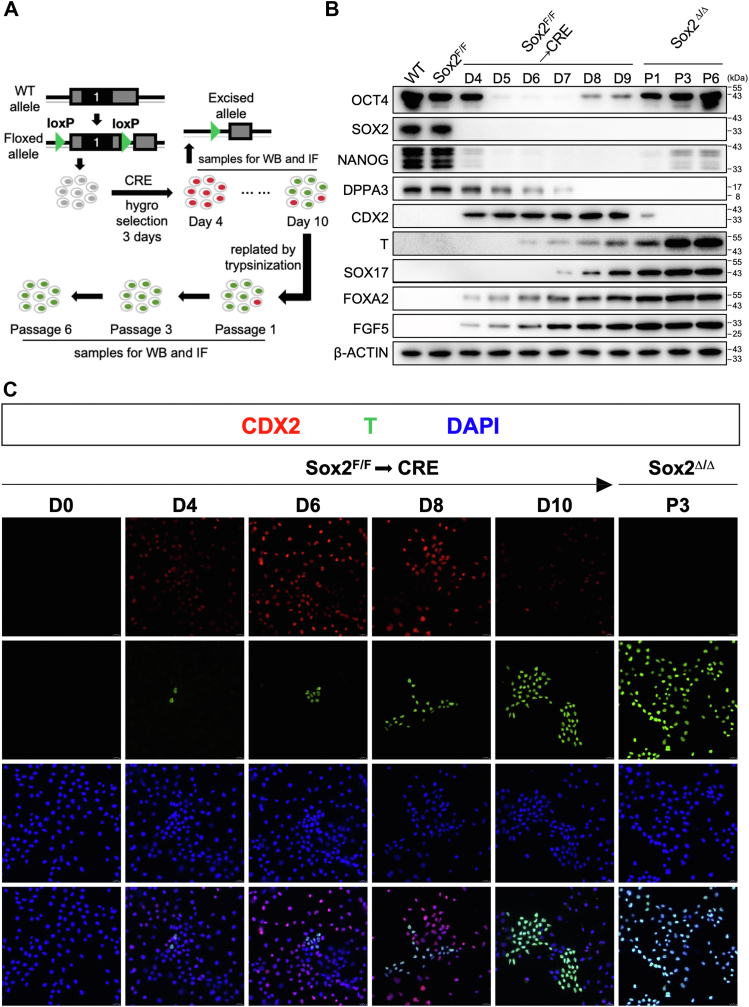
**Sox2 ablation results in dynamic changes in gene expression characteristics of postimplantation epiblasts and trophoblast lineages**. *A*, The schematic diagram illustrating the chronological sequence and critical stages of the experimental procedure. *B*, Western blot for selected proteins in Sox2^F/F^ ESCs following lenti-Cre infection. β-Actin served as a loading control. C, Immunofluorescence analysis for CDX2 (*red*), T (*green*), or DAPI (*blue*) in Sox2^F/F^ or Sox2^F/F^ ESCs following lenti-Cre infection. The images were captured using confocal microscopy with a 63x objective lens. Merge, merged images. D, day; P, passage.

The moderate induction of the typical EpiSC marker T achieved by ablating Sox2 in ESCs prompted us to examine the temporal expression of a panel of genes characteristic of epiblast lineages along with a number of TE- and pluripotency-associated markers in Sox2^F/F^ ESCs following lenti-Cre infection (Fig. 4*A*). Western blot analysis revealed that Cdx2 was easily detected as early as 4 days after Cre infection and plateaued around day 6 (Fig. 4*B*). The transition from naïve to primed state initiated as early as day 4 with gradually increased expression of Foxa2 and Fgf5, closely followed by expression of T and SOX17. In parallel with the expression of those lineage-defining markers, protein levels of pluripotency factors Oct4, Nanog, and Dppa3 declined following Sox2 deletion in ESCs (Fig. 4*B*), indicating a progression toward differentiation. Interestingly, Oct4 was decreased rapidly to the lowest level at day 7 and gradually recovered thereafter, suggesting pluripotent cells exist during culture. The naïve-to-primed transition of Sox2^Δ/Δ^ cells was also monitored by immunofluorescence microscopy to detect the expression profile of epiblast marker T alongside TE marker CDX2 (Fig. 4*C*). In Sox2^F/F^ ESCs cells, cells positive for T and CDX2 were rarely observed, indicating the undifferentiated nature of the cells. As illustrated in Figure 4*C*, CDX2 was readily detected in the vast majority of cells upon Sox2 depletion. However, we also identified a small population of cells that arose later (at day 4) and expressed T but are negative for CDX2 (Fig. 4*C*). When dissociated to single cells with either collagenase or trypsin, Sox2^Δ/Δ^ T^+^, but not Sox2^Δ/Δ^ CDX2^+^ cells, could be propagated in conventional mouse ESC culture conditions without exogenous Fgf2/Activin A supplementation. Thus, Sox2 maintains ESC identity, primarily by suppressing trophectoderm differentiation and preventing the adoption of features resembling primed pluripotency.

**Figure 5:**
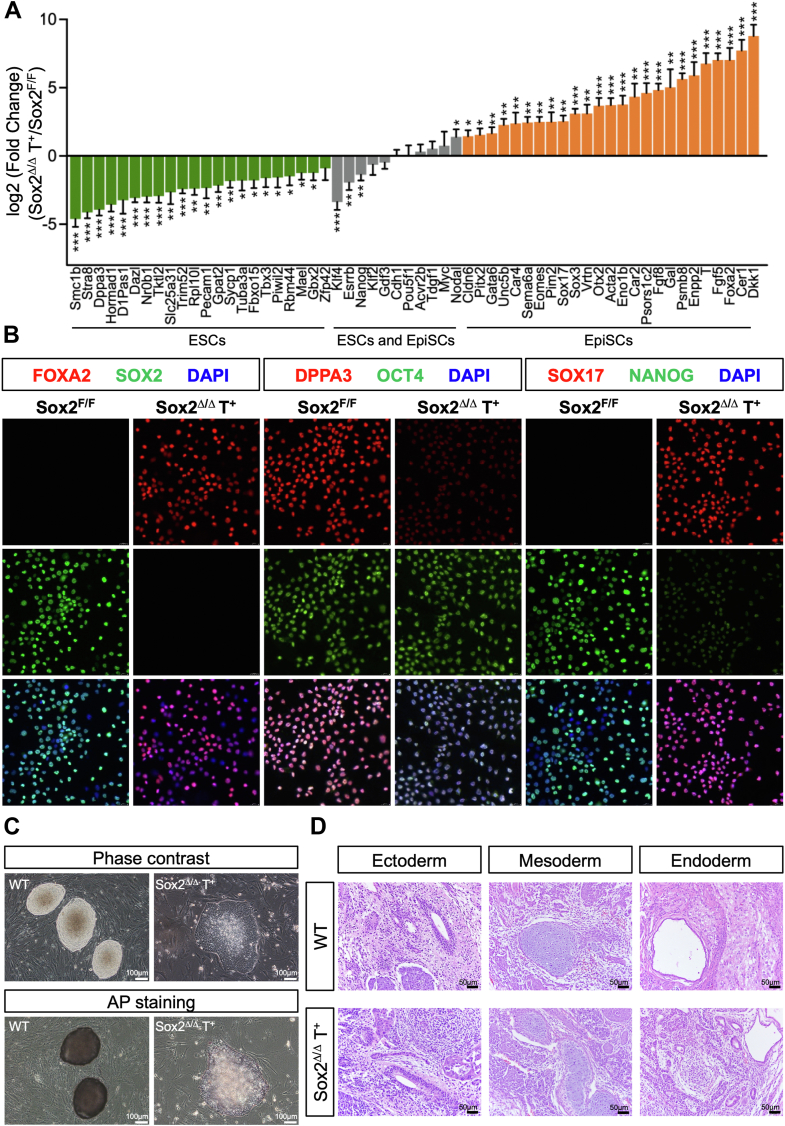
**Sox2-deficient stable cell lines exist in a primed state of pluripotency**. *A*, RT–qPCR analysis of the expression of pluripotency markers specific for ESCs and/or in Sox2^Δ/Δ^ T+ cells. All data are normalized to β-actin and shown relative to control ESCs (set at 1.0). Data were obtained from three independent experiments and expressed as the mean ± SD. (∗*p* < 0.05, ∗∗*p* < 0.01, ∗∗∗*p* < 0.001). *B*, immunofluorescence staining of Foxa2, Dppa3, Sox17 (*red, up*), Sox2, Oct4, and Nanog (*green, middle*) in Sox2^F/F^ or Sox2^Δ/Δ^ T+ cells. DAPI (*blue*) was used to visualize cell nuclei. The image of the merged channels is shown at the *bottom*. The images were captured using confocal microscopy with a 63x objective lens. Merge, merged images. *C*, *top*, phase-contrast images of colonies cultured on a layer of MEFs. *Bottom*, representative images of AP staining of colonies of indicated genotypes cultured on a feeder layer of MEFs. Scale bar, 100 μm. *D*, representative images of tissues of all three germ layers, including gut epithelium (endoderm), cartilage (mesoderm), and neural rosette (ectoderm), from H&E staining of teratomas generated from ESCs of the indicated genotypes. Shown is a representative of three injected mice. Scale bars, 50 μm.

We observed 2665 (1656 up, and 1009 down) differentially expressed genes in Sox2^Δ/Δ^ T^+^ cells compared with Sox2^F/F^ cells (Table S6). Those genes highly correlated and overlapped with those altered in the established EpiSC lines (Fig. S4*B*) (24). GO analysis on the upregulated differentially expressed genes showed that these genes were primarily linked to developmental processes, including embryonic morphogenesis, pattern specification process, and cell fate commitment (Fig. S4*C*). In contrast, downregulated genes were specifically enriched for genes associated with the meiotic cell cycle, embryonic pattern specification, and pathways regulating the pluripotency of stem cells. Analysis of Sox2^Δ/Δ^ T^+^ cells for expression of ESC-specific markers, such as Oct4 and Nanog, showed that Oct4 was expressed at comparable levels in Sox2^F/F^ cells, while Nanog was strikingly underexpressed (Fig. 4*A*). Naive pluripotency-associated genes including Klf4, Nr0b1, Esrrb, which were highly expressed in ESCs, were either not significantly changed (Cdh1 and Acvr2b) or decreased (Klf4, Klf2, Esrrb, Nr0b1, Tbx3, and Gbx2) in Sox2^Δ/Δ^ T^+^ cells. Genes involved in the germ cell differentiation, such as Dppa3, Pecam1, Stra8, and Dazl, which are commonly expressed in ESCs, significantly decreased or not detected in Sox2^Δ/Δ^ T^+^ cells (Fig. 4*A*). On the other hand, transcripts coding for Fgf5, Fgf8, Nodal, T, Foxa2, Gata6, Sox17 and Cer1, genes specifically expressed in the late epiblast and early germ layers, were expressed at higher levels in Sox2^Δ/Δ^ T^+^ cells, in contrast to their low levels in ESCs (Fig. 5, *A* and *B*). Therefore, Sox2^Δ/Δ^ T^+^ cells share a gene expression program reminiscent of EpiSCs, rather than that of ESCs.

Consistent with the gene expression profile, the CpG islands in the promoter regions of the Oct4 gene were almost completely unmethylated in both Sox2 ^F/F^ and Sox2^Δ/Δ^ T^+^ (Fig. S4*D*) (25). However, the promoter regions of Dppa3 were hypomethylated in Sox2 ^F/F^ ESCs but heavily methylated in Sox2^Δ/Δ^ T^+^ cells (26). In addition, two cis-regulatory elements, the distal and proximal enhancers (DE and PE), differentially control expression of the Oct4 gene in the ICM (ESCs) and postimplantation epiblast (EpiSCs), respectively (6, 27). Sox2^Δ/Δ^ T^+^ maintained in ESC medium showed strong activity in the Oct4-proximal enhancer PE, in contrast with Sox2^F/F^ ESCs, which mainly exhibit activity in the DE, (Fig. S4*E*). The Sox2^Δ/Δ^ T^+^ cells were assessed for their ability to form colonies after seeding on a feeder layer. After 5 to 7 days, most cultures revealed large, flattened colonies having weak AP activity, which generally marks pluripotent stem cells such as mouse ESCs (Fig. 5*C*). Of note, we obtained very similar results with 129-derived ESCs (E14Tg2a), thereby suggesting a general phenomenon (Figs. S5, *A*–*G* and S6*A*). Thus, Sox2^Δ/Δ^ T^+^ cells share common features of EpiSCs with respect to gene expression patterns, morphology, and epigenetic profiles.

To further determine the pluripotency of Sox2^Δ/Δ^ T^+^ cells, these cells were subcutaneously injected into immunocompromised mice to test the efficiency of teratoma formation. Remarkably, Sox2^Δ/Δ^ T^+^ cells efficiently formed teratomas consisting of multilineage tissues derived from all three germ layers (Fig. 5*D*). Extensive differentiation was evident and included cartilage, muscle, neural rosettes, gastrointestinal epithelium, and adipocytes among others. Taken together, our findings support the notion that, despite the presence of LIF and other growth factors from the serum or secreted by the feeders, Sox2^Δ/Δ^ T^+^ cells adapt a pluripotent state that highly resembles EpiSCs.

### Autocrine Fgf and Nodal signaling are required for the acquisition and maintenance of Sox2^Δ/Δ^ T^+^ cells

**Figure 6:**
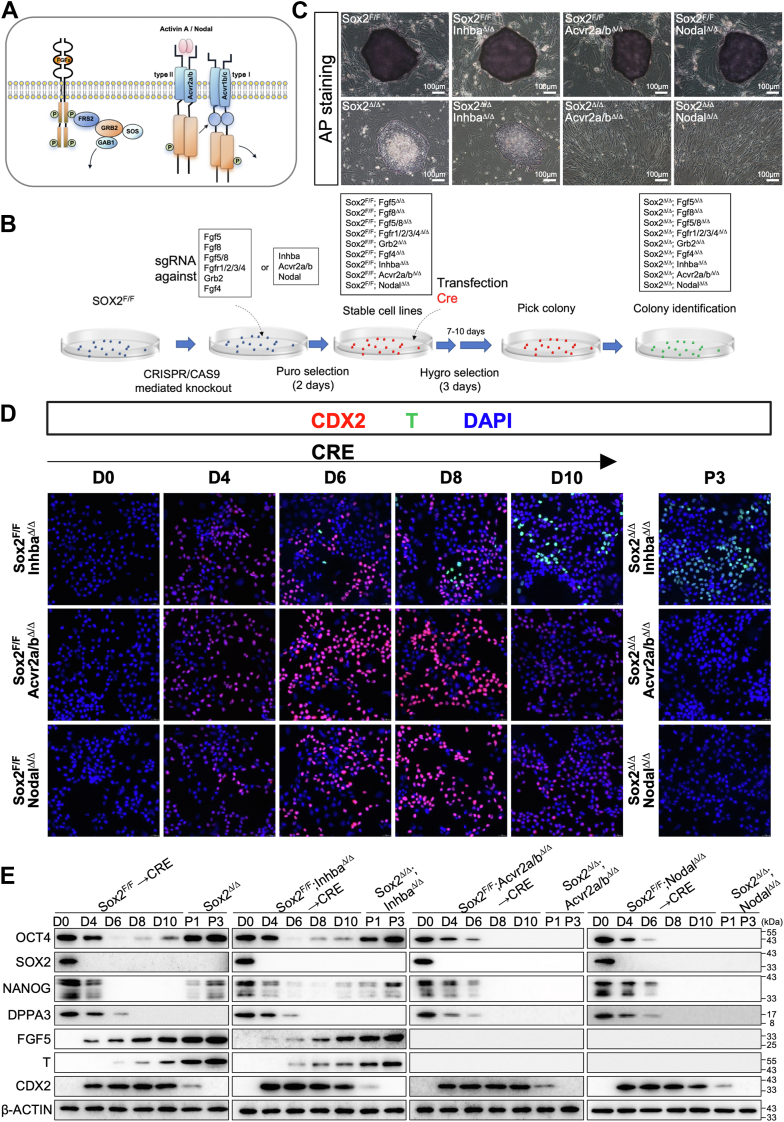
**Autocrine Nodal signaling is required for the acquisition and maintenance of Sox2^Δ/Δ^ T^+^ cells.***A*, schematic model of the Fgf and Activin signaling cascade in ESCs. *B*, schematic representation of experimental procedures. *C*, representative images of AP staining of colonies of indicated genotypes cultured on a feeder layer of MEFs. Scale bar, 100 μm. *D*, immunofluorescence analysis for CDX2 (*red*), T (*green*), or DAPI (*blue*) in ESCs of indicated genotypes following lenti-Cre infection. All images were shown merged with the *green* and *red* fluorescent channels. The images were captured using confocal microscopy with a 63x objective lens. *E*, Western blot analysis of indicated protein levels in cells with indicated genotypes. β-Actin served as a loading control. D, day; P, passage.

It is well established that EpiSCs rely on Fgf and Nodal/Activin signaling to maintain their undifferentiated state (Fig. 6*A*). However, Sox2^Δ/Δ^ T^+^ cells could be obtained and stably propagated in an ESC medium without exogenous Fgf and Activin A. This prompted us to explore the role of these two signaling pathways in Sox2-mediated naïve-primed transition. To determine if signaling from Nodal/Activin receptor family is essential for the phenotypes observed in Sox2 null cells, we generated knockout of Inhba (the gene encoding Activin A), Nodal, or double knockout of Activin type II receptors A and B (Acvr2a/b) ESCs harboring floxed alleles of Sox2 (Sox2^F/F^) (Figs. 6*B* and S6*B*). These mutant ESCs did not exhibit any overt phenotype, demonstrated strong AP staining and formed compact colonies morphologically indistinguishable from Sox2^F/F^ (Figs. 6*C* and S6*C*). Next, these mutants harboring floxed alleles of Sox2 were infected with lenti-Cre. Surprisingly, following removal of Sox2 in addition to Inhba, the extent of EpiSC-specific gene activation was comparable to that of Sox2^Δ/Δ^ cells, as evidenced by immunofluorescence and Western blot (Fig. 6, *D* and *E*), and these mutant cells displayed the flattened two-dimensional colony morphology similar to that of the Sox2^Δ/Δ^ T^+^ cells indicative of the naïve-to-primed transition (Fig. S6*C*). However, we observed a complete loss of EpiSC-specific gene activation following the removal of Sox2 in addition to Nodal or Acvr2a/b (Fig. 6, *C*–*E*). In addition, Sox2^Δ/Δ^; Nodal^Δ/Δ^ and Sox2^Δ/Δ^; Acvr2a/b^Δ/Δ^ cells lost the ability to form colonies on feeder layers (Figs. 6*C* and S6*C*). This indicates that the acquisition and maintenance of primed pluripotency in Sox2^Δ/Δ^ T^+^ cells strongly depend on Nodal rather than Activin A signaling, which is consistent with an increase in Nodal levels in these cells (Fig. 5*A*).

**Figure 7:**
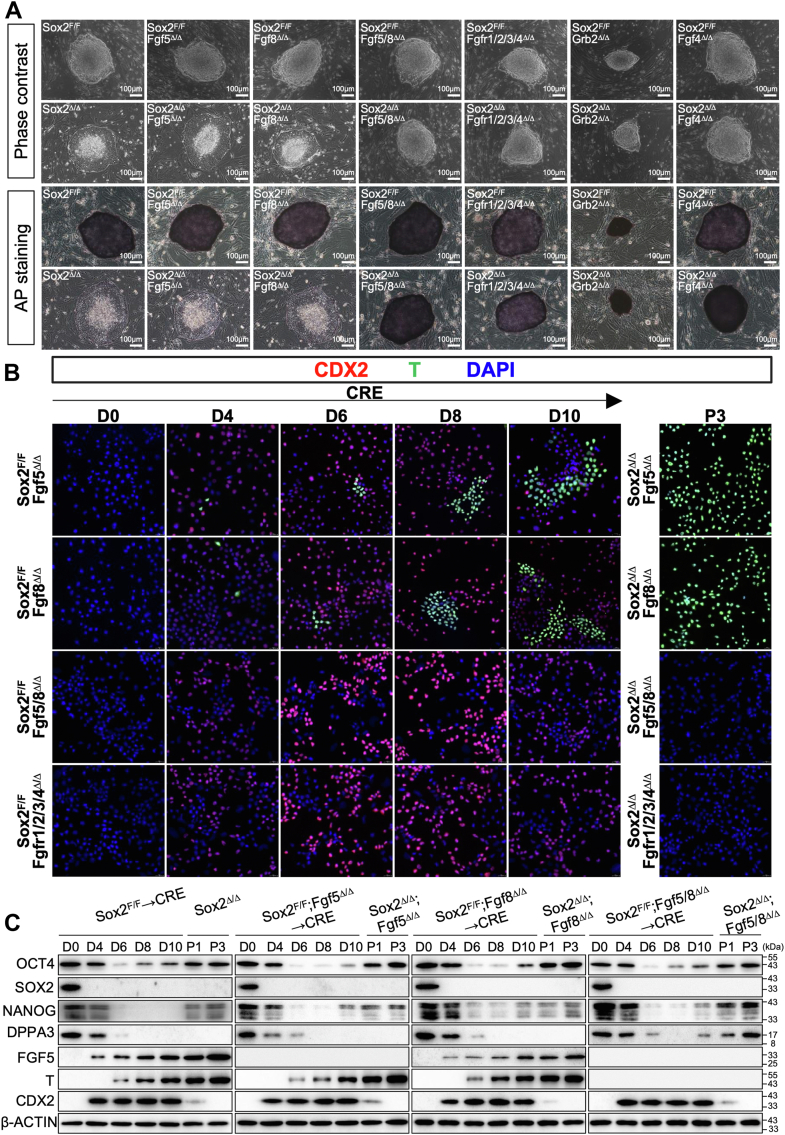
**Signaling by Fgf is of critical importance in the acquisition and maintenance of Sox2^Δ/Δ^ T^+^ cells**. *A*, *Top*, phase-contrast images of colonies cultured on a layer of MEFs. *Bottom*, representative images of AP staining of colonies of indicated genotypes cultured on a feeder layer of MEFs. Scale bar, 100 μm. *B*, Immunofluorescence analysis for CDX2 (*red*), T (*green*), or DAPI (*blue*) in ESCs of indicated genotypes following lenti-Cre infection. All images were shown merged with the *green* and *red* fluorescent channels. The images were captured using confocal microscopy with a 63x objective lens. *C*, Western blot analysis of indicated protein levels in cells with indicated genotypes. β-Actin served as a loading control. D, day; P, passage.

To select the most prominent Fgfs among all 22 members of the Fgf family, we first performed qRT-PCR to analyze the expression of all Fgf genes in Sox2^Δ/Δ^ T^+^ cells. Compared with their parental counterparts, Sox2^Δ/Δ^ T^+^ cells displayed a slight increase in Fgf15 and a dramatic increase in Fgf5 and Fgf8 (Fig. S7*A*). Notably, ablation of Sox2 in ESCs resulted in a reduction of mRNA levels of Fgfr3/4, but not Fgfr1/2. We then generated single or combined knockout of Fgf5, Fgf8, Fgf receptors1–4 (Fgfr1–4) and Grb2, which is an adaptor protein required for signaling downstream of Fgf receptors, ESCs harboring floxed alleles of Sox2 (Sox2^F/F^) (Figs. S7*B* and S8*A*). These conditional mutants retained characteristics typical for undifferentiated ESCs, except Sox2^F/F^; and Grb2^Δ/Δ^, which displayed a low proliferation rate (Fig. 7, *A*–*C*). Interestingly, these mutants also displayed an elevated level of Nanog, suggesting a close association between Fgf signaling and Nanog repression in ESCs (Fig. 7*C*). As shown in Figure 7, *B* and *C*, loss of Fgf5 or Fgf8 in Sox2^Δ/Δ^ ESCs resulted in a robust induction in the expression of EpiSC-specific markers, and these compound mutants appeared to be flat in colony morphology similar to that of the Sox2^Δ/Δ^ T^+^ cells, suggesting the naïve-to-primed transition. Astonishingly, Sox2^Δ/Δ^; Fgf5/8^Δ/Δ^ cells formed dome-shaped colonies that morphologically resembled the control undifferentiated ESCs (Fig. 7*A*). Additionally, combined knockout of Fgf5/8 in Sox2^Δ/Δ^ completely eliminated the up-regulation of the EpiSC-specific markers, consistent with retention of pluripotency markers Oct4, Nanog, and Dppa3 (Fig. 7*C*), suggesting that there exists a significant level of functional redundancy between Fgf5 and Fgf8 during the transition from naive to primed pluripotency. In line with these findings, the phenotypes of Sox2^Δ/Δ^;Fgf5/8^Δ/Δ^ were shared with Sox2^Δ/Δ^; Fgfr1-4^Δ/Δ^ and Sox2^Δ/Δ^; Grb2^Δ/Δ^, indicating a rescue of the naive to primed conversion in the absence of Fgf5/8, Fgfr1-4, or Grb2 (Fig. 7, *A*–*C* and S8*B*). Notably, we found that Fgf4, which is the most highly expressed paralog in the Fgf gene family in undifferentiated ESCs, was also required for the naïve-primed state conversion in Sox2^Δ/Δ^ T^+^ cells (Figs. 7*A*, S8, *B* and *C*). This is consistent with previous observations that Fgf4-deficient ESCs are refractory to differentiation (28). Therefore, autocrine Fgf and Nodal are vital components of signaling pathways that control the transition from naïve to primed pluripotency in Sox2-null cells.

### Sox3 compensates for the function of Sox2 in the maintenance of primed pluripotency

**Figure 8:**
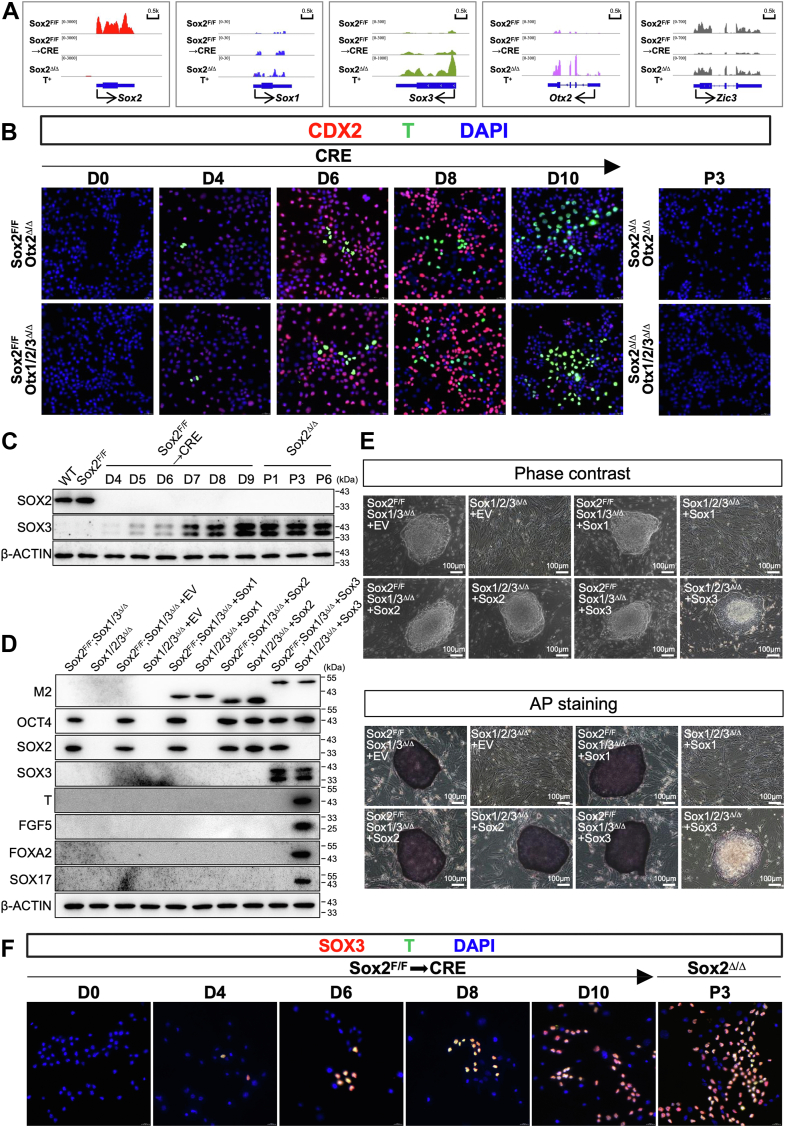
**Sox3 compensates for the function of Sox2 in the maintenance of primed pluripotency**. *A*, representative genome browser screenshots of RNA-seq tracks illustrating RNA expression of the corresponding genes in cells of indicated genotypes. *B*, immunofluorescence analysis for CDX2 (*red*), T (*green*) or DAPI (*blue*) in ESCs of indicated genotypes following lenti-Cre infection. All images were shown merged with the *green* and *red* fluorescent channel. The images were captured using confocal microscopy with a 63x objective lens. *C*, Western blot for Sox2 and Sox3 in Sox2^F/F^ ESCs following lenti-Cre infection. β-Actin served as a loading control. *D*, Western blot for selected proteins in cells with indicated genotypes. β-Actin served as a loading control. *E*, *Top*, phase-contrast images of colonies cultured on a layer of MEFs. *Bottom*, Representative images of AP staining of colonies of indicated genotypes cultured on a feeder layer of MEFs. Scale bar, 100 μm. EV, empty vector. *F*, immunofluorescence analysis for Sox3 (*red*), T (*green*), or DAPI (*blue*) in ESCs of indicated genotypes following lenti-Cre infection. All images were shown merged with the *green* and *red* fluorescent channels. The images were captured using confocal microscopy with a 63x objective lens. D, day; P, passage.

Probing deeper into the molecular mechanisms of coordinating the naïve to primed transition, we tested whether the derepression of primed pluripotency-associated transcription factors led to the cell fate conversion observed in Sox2^Δ/Δ^ T^+^ cells. In Sox2^Δ/Δ^ T^+^ cells, we observed dramatic upregulation of Otx2, but not Zic2 or Zic3 (Figs. 8*A* and S9*A*), which were recently shown to function during the transition from the naïve to primed states of pluripotency (10, 11). Using the procedure described in Figure 6*B*, we found that, loss of Otx2 in Sox2^Δ/Δ^ ESCs led to a fairly normal expression of T, and these mutant cells grew as large flat colonies (Fig. S9, *B* and *C*). Similarly, induction of EpiSC-specific genes following the removal of Sox2 in addition to Otx1-3 were highly comparable to the changes observed in Sox2^Δ/Δ^;Otx2^Δ/Δ^ cells (Figs. 8*B*, S9, *B* and *C*), suggesting no redundant function within the Otx family with respect to this phenotype. Importantly, after 2 to 3 passages, the great majority of both knockout colonies displayed domed morphology with strong AP staining, almost complete extinction of T^+^ cells (Fig. S9*C*). These findings indicate that Otx2 is required for maintaining the stability of primed pluripotency but not for the initial transition from the naïve to primed states, in agreement with previous observations (10).

It has been well established that Sox2 and Oct4 form heterodimers to cooperatively activate pluripotency-associated genes for the maintenance of naïve pluripotency (21, 23). In addition, coablation of Sox3 and Sox2 resulted in severe phenotypes such as complete loss of pluripotency-associated gene expression that were not observed in single mutants (Fig. 3*E*). Therefore, we hypothesized that Oct4 switches partnering from Sox2 to Sox3 to initiate the transcriptional transition to primed pluripotency in Sox2^Δ/Δ^ T^+^ cells. In support of this idea, RNA-Seq and RT-qPCR analysis revealed that the expression of Sox3,but not Sox1, increased immediately after lenti-Cre infection of Sox2^F/F^ ESCs and reached a peak around day 9 and then remained at that level thereafter (Figs. 8*A* and S9*A*). Western blot analysis over the differentiation period confirmed these dynamics (Fig. 8*C*). In contrast, Otx2 was initially significantly downregulated, maintaining these levels until at least day 8, but heavily elevated by the time the primed state was acquired (Fig. S9*A*). The above-mentioned Sox1/3^Δ/Δ^; Sox2^F/F^ ESCs were used to further address the roles of Sox3 in the acquisition and maintenance of primed pluripotency. As expected, ectopic expression of Sox2 completely rescued the phenotypic defects observed in Sox1-3^Δ/Δ^ cells (Fig. 8, *D* and *E*). The rescued cells formed more compact and domed colonies that resembled ESC colony morphology and expressed AP. Importantly, the transgenic re-expression of Sox3 prevented the further decline in pluripotency protein levels observed in Sox1-3^Δ/Δ^ cells and produced flat colonies of compact cells characteristic of EpiSCs, hallmarked by the expression of epiblast genes like T and many lineage-specific genes (Fgf5, Foxa2, and Sox17). This finding is in line with recent reports indicating that Sox2 and Sox3 operate in a functionally redundant way to maintain the identity of primed EpiSCs (29). The Sox3-expressing Sox1-3-null cells could be passaged and maintained for at least 30 passages without any change in colony morphology (Fig. 8, *D* and *E* and S9*D*). In contrast, ectopic expression of Sox1 failed to rescue the triple mutant phenotype. These findings were further supported by immunofluorescence staining, demonstrating that Sox3 was coexpressed with T, but not Cdx2, during the naïve-to-primed pluripotency transition (Fig. 8*F*). Moreover, similar results were observed when the rescue assay was carried out in Sox2^F/F^ ESCs (Fig. S10, *A* and *B*). Collectively, our findings indicate the functional redundancy between Sox2 and Sox3 in preserving pluripotency in primed EpiSCs and suggest that further studies are needed to delineate the molecular mechanisms of core transcriptional regulatory circuitry in pluripotent Sox2-null cells.

## Discussion

Although both ESCs and EpiSCs exhibit the ability to give rise to all cell types arising from the three germ layers, these two types of pluripotent cells are clearly distinguished by their behavior, morphologies, growth factor requirements, transcriptional and epigenetic profiles, and developmental potential (6, 7). Interestingly, these two states can also be interconverted *in vitro* through the induction of extrinsic signaling or genetic modification (25, 30, 31, 32). EpiSCs can be reverted to naïve cells by ectopic overexpression of a variety of transcription factors associated with naïve pluripotency including Klf4, Klf2, c-Myc, and Nanog, under LIF-containing conditions (25, 31). Notably, a similar conversion of EpiSCs from permissive strains to ESCs is achieved upon exposure to LIF/Stat3 signaling alone (25, 30). Likewise, naïve cells or ICM cells can be gradually converted to a primed state closely resembling EpiSCs by culturing with Activin A and Fgf2 (25, 33). It has been proposed that two pluripotent states can be considered a metastable equilibrium governed by the genetic background where exogenous factors can convert one state into another (25). However, the molecular mechanism controlling the EpiSCs state and naïve-primed pluripotent transition remains to be fully clarified.

To obtain insights into the molecular mechanism underlying the interconversion between naïve and primed pluripotent states, we first performed a CRISPR-based loss-of-function screen in ESCs to detect genes essential for the transition from naïve to primed state. Surprisingly, Sox2 was identified as a candidate factor affecting the transition (Fig. 1). The role of Sox2 in stem cell self-renewal and the acquisition and maintenance of naïve pluripotency is well-documented (9, 20, 34). Like Oct4 knockout mice, mutant mice lacking Sox2 are embryonically lethal due to the failure to form pluripotent ICM (20, 23). Similarly, the elimination of Sox2 in already established ESCs results in the loss of self-renewal and pluripotency, which is coupled with inappropriate differentiation to trophectoderm (23). Indeed, after infection of Sox2^F/F^ ESCs with Cre lentivirus, we observed that the vast majority of infected cells were positive for Cdx2, consistent with the established role of Sox2 in trophoblast development (23) (Fig. 3). However, we also identified a small population of cells that express epiblast marker T but were negative for Cdx2 (Fig. 4).

Sox2^Δ/Δ^ T^+^ cells grew as large flat, monolayer colonies and were thus morphologically distinct from mouse ESCs, which form three-dimensional domed colonies. Sox2 null stable cells showed a very weak AP activity, thus demonstrating their primed pluripotent state (Fig. 5). Although Sox2^Δ/Δ^ T^+^ cells continued Oct4 expression compared to their parental counterpart cells, many pluripotency-associated transcription factors for ESCs, including Nanog, Dppa3, Esrrb, Zfp42, Nr0b1, and Klf4, were absent or expressed at low levels in Sox2^Δ/Δ^ T^+^ cells, strongly suggesting that these cells may possess a distinct regulatory circuitry underlying pluripotency (Fig. 5*A*). In contrast, genes associated with the epiblast and early germ layers, such as Fgf5, T, Otx2, Foxa2, Dkk1, Sox17, and Cer1, were expressed at higher levels in Sox2^Δ/Δ^ T^+^ cells. Sox2^Δ/Δ^ T^+^ cells showed the unique epigenetic states of postimplantation epiblast, including the preferential use of the PE over the DE to drive Oct4 expression and complete DNA methylation at the promoters of a set of pluripotency and germ cell-associated genes, such as Dppa3 (Fig. S4, *D* and *E*). Thus, our results demonstrated that, despite the presence of LIF and other growth factors from the serum or secreted by the feeder layers, stable Sox2-null ESCs adapt to a pluripotent state that closely resembles EpiSCs. Although Sox2^Δ/Δ^ T^+^ cells were generated and cultured without the addition of exogenous Fgf and Activin A, these cells strictly depended on Fgf and Activin signaling for maintaining their primed pluripotency (Figs. 6 and 7). The levels of Nodal, Fgf5 and Fgf8 were dramatically elevated in Sox2^Δ/Δ^ T^+^ cells compared to ESCs (Fig. 5*A*), while Nodal- or Fgf5/8-deficient cells lost the ability to commit to the primed state, strongly suggesting Fgf and Nodal exert their function in an autocrine fashion. Therefore, distinct states of pluripotency are maintained by a synergistic interplay of extrinsic signaling pathways and intrinsic networks of transcription factors. The establishment of stable Sox2-null pluripotent cell lines will enable the elucidation of the precise controls that regulate the maintenance of primed pluripotency and the transition of cells between different states of pluripotency and differentiation.

In the early mouse embryo, preimplantation-to-postimplantation epiblast transition is governed by changes in the pluripotency gene regulatory network. Transcription factors highly expressed in epiblast cells of the ICM, such as Klf4, Klf5, Esrrb, Zfp42, and Tbx3 are undetectable in the epiblast of E7.5 embryos (35, 36, 37, 38). Accordingly, a number of the pluripotency transcription factors downregulated following implantation are also downregulated or eliminated in EpiSCs (6). Consistent with these observations, stable Sox2-null cells displayed little to no expression of the naïve state markers Zfp42, Dppa3, Nr0b1, Gbx2, Klf4, Klf2, and Fgf4 (Fig. 5*A*). Among the core pluripotency factors, Oct4 expression was maintained, whereas Nanog expression was substantially reduced. This is in line with previous observations that the expression levels of Sox2 and Nanog are lower in EpiSCs (32, 39). Sox3, a close paralog of Sox2, was expressed at very low levels in undifferentiated ESCs, but it was highly expressed in the absence of Sox2 (Fig. 8*A*). This indicates that transcriptional activation of Sox3 might functionally compensate for the absence of Sox2 in Sox2^Δ/Δ^ T^+^ cells. This idea was supported by several lines of evidence. First, Sox3 was immediately induced upon Sox2 ablation (Fig. 8*A*), and positively coexpressed with T during Sox2^Δ/Δ^ T^+^ cell development (Fig. 8*F*). Second, the removal of both Sox2 and Sox3 caused a rapid loss of pluripotency (Fig. 3*E*), and the severe defects observed in Sox2/3-deficient ESCs were rescued by ectopic expression of Sox3 (Fig. 8). Finally, Sox3-rescued Sox2/3-null showed similar characteristics to Sox2^Δ/Δ^ T^+^ cells, which shared defining features with mouse EpiSCs (Fig. 8). Notably, as a reprogramming factor, Sox2 can be substituted by either Sox1 or Sox3 (40), and replacement of an endogenous Sox3 allele with a Sox2 open reading frame can restore pituitary and testis defects that occur in Sox3 null mice (41), providing further evidence of the functional equivalence among SoxB1 members. Additionally, multiple studies have shown that EpiSCs show substantially higher levels of Sox3 compared with those in ESCs, while Sox2 mRNA levels in EpiSCs are lower than in ESCs (29, 39, 42, 43, 44), indicating that the balance between Sox2 and Sox3 dictates the maintenance and transition among these pluripotent states. In summary, our results indicate that Sox2 maintains ESC pluripotency not only by preventing trophectoderm differentiation but also by suppressing the transition of naïve cells toward primed pluripotency. Future studies will be needed to identify Sox3 targets and interaction partners during different pluripotent cell state transitions. It will also be of great interest to define the roles of SOXB1 proteins in human stem cell pluripotency.

## Experimental procedures

### Cell lines

Unless otherwise indicated, mouse C57BL/6 and E14Tg2a (from ATCC) ESCs were cultured on gelatin-coated tissue culture plates either feeder-free or with mitomycin C-treated MEFs at 37°C and 5% CO_2_, in DMEM containing 15% ESC-qualified fetal bovine serum, nonessential amino acids, sodium pyruvate, 2 mM L-glutamine, 0.5 mM β-mercaptoethanol, penicillin/streptomycin and 10 ng/ml of LIF as described (45). The ESCs were separated from the MEF feeder cells by short-term (30 min at 37°C) plating of the trypsinized mixed cell population on plates not coated with gelatin. The feeder cells preferentially adhere and the ESCs are readily harvested for further experiments. All cultures were regularly checked to confirm the absence of *mycoplasma* contamination. The ESCs were trypsinized to single cells and subsequently reseeded onto mytomycin-C growth-arrested MEF feeder cells. The medium was changed every other day. Cell colonies were photographed under an inverted phase contrast microscope after 7 days in culture. Of note, colonies exhibiting a pronounced growth defect were photographed on day 12.

### Cloning and plasmid constructs

Full-length cDNAs encoding Sox1, Sox2, and Sox3 were amplified by RT–PCR from mouse mRNA and cloned into lentivirus vector with a FLAG epitope tag (DYKDDDDK) by standard DNA cloning methods. All the constructed plasmids were confirmed by DNA sequencing. Lentiviral production and infection were performed as described previously (45).

### CRISPR/Cas9-mediated genome engineering

The single-guide RNAs (sgRNAs) targeting the region of interest were designed by an online CRISPR design tool (http://crispor.tefor.net/). A pair of oligonucleotides for each sgRNA were annealed and cloned into the BbsI-digested Cas9 and sgRNA-expressing PX459 vector (Addgene plasmid ID: 62,988). sgRNA-expressing constructs were verified by DNA sequencing. For the detailed sgRNA sequences for each gene, see supplementary experimental procedures. Targeting donor constructs with appropriate homology arms flanking the insertion sequence were generated by standard molecular cloning methods (45). ESCs were transfected with 1 μg of each Cas9 guide described above, and 1.5 μg of linearized targeting construct (where appropriate) using Lipofectamine 2000 (ThermoFisher) according to manufacturer’s guidelines. After transfection for 24 h, cells were subjected to puromycin (2 μg/ml) selection for 48 h to selectively remove any non-transfected cells. Approximately 1 week after seeding ESCs on feeder MEFs, individual clones were manually picked into 24-well plates and expanded for genomic DNA extraction and continued culture. The final colonies are screened by PCR for the presence of the desired genome modification. All primers are listed in Table S7.

### EB formation and analysis

EB formation from ESCs or was induced using the hanging drop method modified from previously described (45). Briefly, cells were harvested by trypsin or collagenase digestion and resuspended in IMDM medium without LIF (nonessential amino acids, iron saturated holo-transferrin (200 μg/ml), ascorbic acid (50 μg/ml), 2 mM L-glutamine, sodium pyruvate, 450 μM monothioglycerol, penicillin/streptomycin, and 15% ES cell–grade serum). The cells were seeded at a density of 500 cells per 30 μl drop in the lids of petri dishes for 3 days. Then, the formed EBs were transferred to uncoated Petri dishes and incubated on a rotating shaker at 60 rpm in 5% CO_2_ at 37°C. EBs were photographed or harvested at different time points. Total RNA was extracted from EBs using TRIzol reagent (Invitrogen) according to the manufacturer’s instructions and analyzed by RT-PCR. The levels of marker genes representative of the undifferentiated state and the three embryonic germ layers were examined to evaluate their *in vitro* differentiation potential. The mRNA of β-actin was analyzed as an internal control. Primer details are given in Table S7.

### RNA preparation and real-time qPCR

Cells were first rinsed in 1x phosphate-buffered saline (PBS) and total RNA was extracted using a TRIzol reagent (Invitrogen), according to the manufacturer's protocols. First-strand cDNA was synthesized using HiScript II first Strand cDNA Synthesis Kit (Vazyme) and oligo(dT) as a primer. Reaction mixtures without reverse transcriptase were used as negative controls for genomic DNA contamination. All quantifications were performed in triplicate using PowerUp SYBR Green Master Mix (Applied Biosystems) with the StepOnePlus Real-Time PCR System, according to the manufacturer’s protocol. Relative expression levels of each gene normalized to β-actin were calculated as 2^−ΔCt^, where ΔCt = Ct _detected gene_ – Ct_β-actin_. Statistical analysis for the comparison between samples was performed using Student’s *t* test. All primers are listed in Table S7.

### Alkaline phosphatase staining

The cells of interest were digested with trypsin or collagenase and then cultured on mitomycin C-treated MEFs in an appropriate medium for 7 to 12 days to form colonies, which were washed with phosphate-buffered saline (PBS) twice and fixed with 4% paraformaldehyde for 2 min followed by washing twice with PBS, and then incubated in freshly prepared staining solution following the protocol recommended by the manufacturer. Finally, the stained colonies were photographed under a microscope.

### Western blot and immunofluorescence staining

Cells were lysed on ice with RIPA buffer [50 mM tris-HCl (pH 8.0), 1% Triton X-100, 0.1% SDS, 150 mM NaCl, 1 mM EDTA, 0.5% sodium deoxycholate supplemented with fresh 2 mM Na_3_VO_4_, 100 mM NaF, 1 mM dithiothreitol, phenylmethylsulfonyl fluoride (10 mg/ml), and Protease Inhibitor Mix (Sigma-Aldrich)] for 30 min, and the supernatant was boiled in SDS sample buffer for 5 min at 100°C, subjected to SDS-PAGE, and transferred to a PVDF membrane (Millipore). Membranes were blocked with 5% skimmed milk in PBST and subsequently probed with the primary antibodies in blocking solution at 4°C overnight, washed and incubated with HRP-conjugated secondary antibody, and visualized using an enhanced chemiluminescence detection kit.

### Cells cultured on 0.1% gelatin-coated coverslips were fixed with 4% paraformaldehyde

For 10 min at room temperature, followed by permeabilization with 0.5% Triton X-100 in PBS, and subsequently incubated with blocking buffer (5% bovine serum albumin in PBS). After blocking, the samples were incubated overnight at 4°C in a humidity chamber with a mixture of primary antibodies diluted in the blocking solution. Slides were washed with PBS followed by incubation with the appropriate secondary antibody conjugated with Alexa Flour 488 or 594 dyes (1:150 dilution) for 2 h in the dark. For DNA staining, the slides were incubated in diluted DAPI (4′-6-diamidino-2-phenylindole) solution in PBS for 10 min. Fluorescent images were obtained and analyzed using a Zeiss 880 laser scanning confocal microscope.

### Teratoma formation assay

The experimental animal facility has been accredited by the Association for Assessment and Accreditation of Laboratory Animal Care International, and the Institutional Animal Care and Use Committee of the Model Animal Research Center of Nanjing University approved all animal protocols used in this study. Teratoma formation assay was performed as previously described with minor modifications (45). Briefly, the cells were harvested by trypsin (ESCs) or collagenase, followed by incubation on gelatin-coated dishes for 30 min. Non-adherent cells consisting mainly of ESCs were used for further experiments. The cells were washed twice with PBS, resuspended in PBS and then injected subcutaneously into the bilateral flanks of nude mice at 6 weeks of age. Teratomas were allowed to develop for 3 to 10 weeks and were then excised and fixed in 4% neutral buffered paraformaldehyde and dehydrated through a graded series of alcohols to xylene. The tissue was embedded in paraffin, serially sectioned at 5 μm and processed for H & E (haematoxylin and eosin) staining or immunohistochemistry.

### Flow cytometry

#### Cell cycle analysis

Cells were harvested by trypsin or collagenase digestion, washed twice with cold PBS, and then fixed using cold 70% ethanol at −20°C over 24 h. Subsequently, cells were washed twice with PBS, and incubated with DNA staining solution (50 mg/ml propidium iodide and 200 μg/ml RNase A) for 30 min at room temperature followed by analysis on a LSRFortessa flow cytometer equipped with Cell Quest software (BD Biosciences) as described (45).

#### Apoptosis assay

For apoptosis analysis, cells were harvested by trypsin or collagenase digestion, washed twice with cold PBS, stained with fluorescein isothiocyanate–Annexin V (BD Pharmingen) containing propidium iodide (PI) in the dark at room temperature, and analyzed by flow cytometry as described (45).

#### Luciferase reporter assay

Constructs encoding the two cis-enhancer elements (DE and PE) within the Oct4 locus cloned into the pGL3-Promoter Vector (Promega) were utilized to quantify relative Oct4 enhancer activity (6). Plasmids were co-transfected with the Renilla vector (as a control for normalizing luciferase activity), using Lipofectamine 2000. Dual Luciferase Assay (Promega) was performed 2 days later, following the manufacturer’s instructions. The basal activity of the empty luciferase vector was set as 1.0.

#### Bisulfite sequence analysis

To determine the DNA methylation status of Sox2-null cells, genomic DNA was treated with sodium bisulfite, which converts all unmethylated cytosine to uracil, leaving methylated cytosines unchanged, using EpiTect Bisulfite Kit (QIAGEN) according to the manufacturer’s instructions. Oct4 and Dppa3 promoter regions were then amplified using nested primers as previously reported (25, 26). The amplified gene fragments were then separately cloned into a pGEM-T easy vector (Promega) for sequencing and 10 randomly selected clones were sequenced with T7 forward and SP6 reverse primers.

#### RNA sequencing

The total RNA was extracted with TRIzol reagent (Invitrogen) according to the manufacturer’s instructions, and potential contaminating genomic DNA was digested with DNase I (Takara). Multiplexed Illumina sequencing libraries were prepared using the TruSeq Stranded Total RNA Library Prep Gold (Illumina, 20,020,598) following the manufacturer's protocol, and 150 bp paired-end reads were generated with HiSeq3000 sequencer (Illumina) with a depth of 50 million reads per sample. Analysis for differential gene expression was performed using the R package DESeq2 v1.20 with default parameters. Genes were considered to be differentially expressed if an absolute log2 fold change >1 and an FDR smaller than 0.05 were observed. Three biological replicates of each sample were sequenced. The RNA-seq data were deposited at Gene Expression Omnibus under accession number GSE270036 (reviewer token: yfwbekoodncjrwx) and GSE291393 (reviewer token: ubchkgiyffejlkh).

#### Statistics

The results are expressed as means ± SEM in at least three independent experiments unless differently specified. Statistical analysis was performed with GraphPad Prism nine to determine significant differences based on a two-tailed distribution using an unpaired Student’s *t* test, and a probability value (P) of less than 0.05, 0.01, or 0.001 was considered statistically significant and represented as ∗, ∗∗, and ∗∗∗, respectively (∗*p* < 0.05, ∗∗*p* < 0.01, ∗∗∗*p* < 0.001).

## Data availability

The data that support the findings of this study are available upon reasonable request from the corresponding author.

## Supporting information

This article contains supporting information.

## Conflict of interest

The authors declare that they have no conflicts of interest with the contents of this article.
