# Supplementary material for: Loss of sex-determining region Y-box 2 (Sox2) captures embryonic stem cells in a primed pluripotent state
Source: J Biol Chem. 2025 Apr 9;301(5):108501. doi: 10.1016/j.jbc.2025.108501 (PMC12135381; doi:10.1016/j.jbc.2025.108501)
Supplement: Supporting Information [file mmc1.pdf]

Loss of sex-determining region Y-box 2 (SOX2) captures embryonic stem cells in a primed pluripotent state

Min Qi,[1,5] Bowen Wang,[1,5] Huaqi Liao,[1,5] Yuzhuo Xu,[1,5] Lixia Dong,[1] Lijun Xu,[1] Yin Xia,[2] Xiaochun Jiang,[3,*] Shizhang Ling,[3,*] and Jinzhong Qin[1,4,6,*]

*Correspondence author. Jinzhong Qin, Email: qinjz@nju.edu.cn (J.Q.);
Shizhang Ling, Email:lingsz@hotmail.com (S.L.);
Xiaochun Jiang, Email: jiangxiaochun2001@hotmail.com (X.J.)

The PDF file includes:

Figures. S1 to S10

Other Supplementary Material for this manuscript includes the following:
Tables S1 to S7

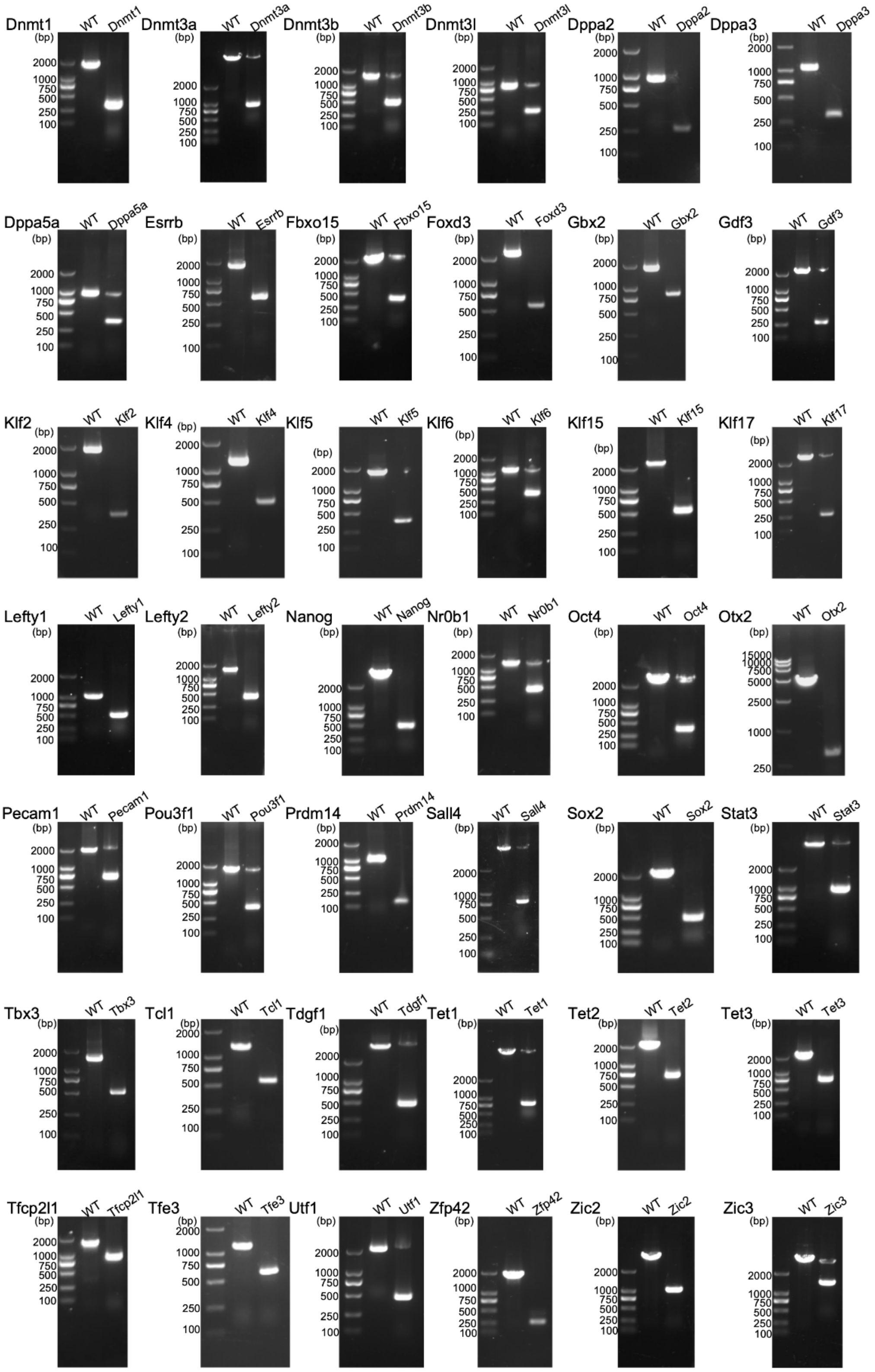
Dnmt1 WT Dnmt1
Dnmt3a WT Dnmt3a
Dnmt3b WT Dnmt3b
Dnmt3l WT Dnmt3l
Dppa2 WT Dppa2
Dppa3 WT Dppa3
Dppa5a WT Dppa5a
Esrrb WT Esrrb
Fbxo15 WT Fbxo15
Foxd3 WT Foxd3
Gbx2 WT Gbx2
Gdf3 WT Gdf3
Klf2 WT Klf2
Klf4 WT Klf4
Klf5 WT Klf5
Klf6 WT Klf6
Klf15 WT Klf15
Klf17 WT Klf17
Lefty1 WT Lefty1
Lefty2 WT Lefty2
Nanog WT Nanog
Nr0b1 WT Nr0b1
Oct4 WT Oct4
Otx2 WT Otx2
Pecam1 WT Pecam1
Pou3f1 WT Pou3f1
Prdm14 WT Prdm14
Sall4 WT Sall4
Sox2 WT Sox2
Stat3 WT Stat3
Tbx3 WT Tbx3
Tcl1 WT Tcl1
Tdgf1 WT Tdgf1
Tet1 WT Tet1
Tet2 WT Tet2
Tet3 WT Tet3
Tfcp2l1 WT Tfcp2l1
Tfe3 WT Tfe3
Utf1 WT Utf1
Zfp42 WT Zfp42
Zic2 WT Zic2
Zic3 WT Zic3
(bp)

Figure S1. CRISPR/Cas9-mediated screening for the molecular regulators that are critical for the naïve-to-primed pluripotent transition. For each gene, two different pairs of sgRNAs targeting the desired regions were designed and cloned into a bicistronic expression vector px459. ESCs were transfected with sgRNA plasmids using Lipofectamine 2000. The day after transfection, ESCs were exposed to puromycin at a concentration of 2 μg/mL for 48 h to eliminate any non-transfected cells. Subsequently, protein and mRNA expression of Dppa3 and T were examined by Western blot and quantitative real-time RT-PCR, respectively. The cleavage efficiency of selected sgRNAs for each gene was validated by PCR analysis of genomic DNA. Shown are agarose gels of genomic PCR products from ESCs co-transfected with a pair of sgRNAs targeting the indicated genes with the higher targeting efficiency.

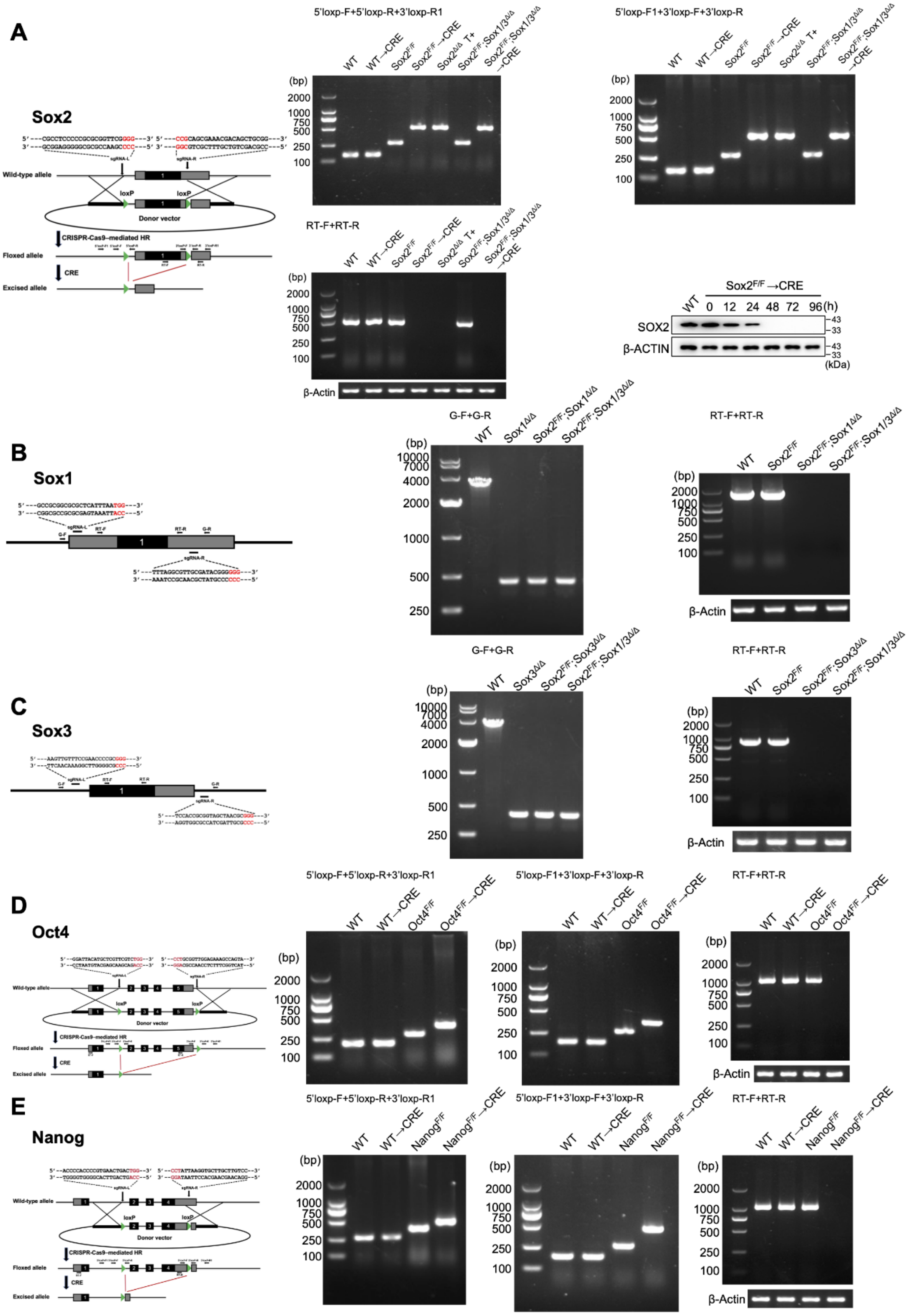
A
Sox2
5'loxp-F+5'loxp-R+3'loxp-R1
5'loxp-F1+3'loxp-F+3'loxp-R
RT-F+RT-R
WT
WT→CRE
Sox2F/F
Sox2F/F→CRE
Sox2Δ/Δ T+
Sox2F/F;Sox1/3Δ/Δ
Sox2F/F;Sox1/3Δ/Δ →CRE
(bp)
2000
1000
750
500
250
100
β-Actin
Sox2F/F →CRE
0 12 24 48 72 96(h)
SOX2
β-ACTIN
43
33
(kDa)
Wild-type allele
Donor vector
loxP
CRISPR-Cas9-mediated HR
Floxed allele
CRE
Excised allele
B
Sox1
G-F+G-R
RT-F+RT-R
Sox1Δ/Δ
Sox2F/F;Sox1Δ/Δ
Sox2F/F;Sox1/3Δ/Δ
10000
7000
4000
C
Sox3
Sox3Δ/Δ
Sox2F/F;Sox3Δ/Δ
D
Oct4
Oct4F/F
Oct4F/F→CRE
E
Nanog
NanogF/F
NanogF/F→CRE

Figure S2. Generation of ESCs deficient in Soxb1 family genes by CRISPR/Cas9-mediated gene targeting. A-E. Left, schematic representation of CRISPR/Cas9 mediated knockout approaches to generate ESCs deficient in Soxb1 family (A-C), Oct4 (D), and Nanog (E). PAM sequences are in red following the sgRNA sequence. The locations of primers used in genomic PCR and RT-PCR are shown. Middle, genotyping of ESCs with indicated gene deletions using primers located upstream and downstream of the deleted region. Right, RT-PCR analysis revealed undetectable transcripts in the indicated mutants. β-actin was used as an internal control. The absence of the Sox2 protein is confirmed by using Western blot (A, right bottom). β-actin acted as a loading control.

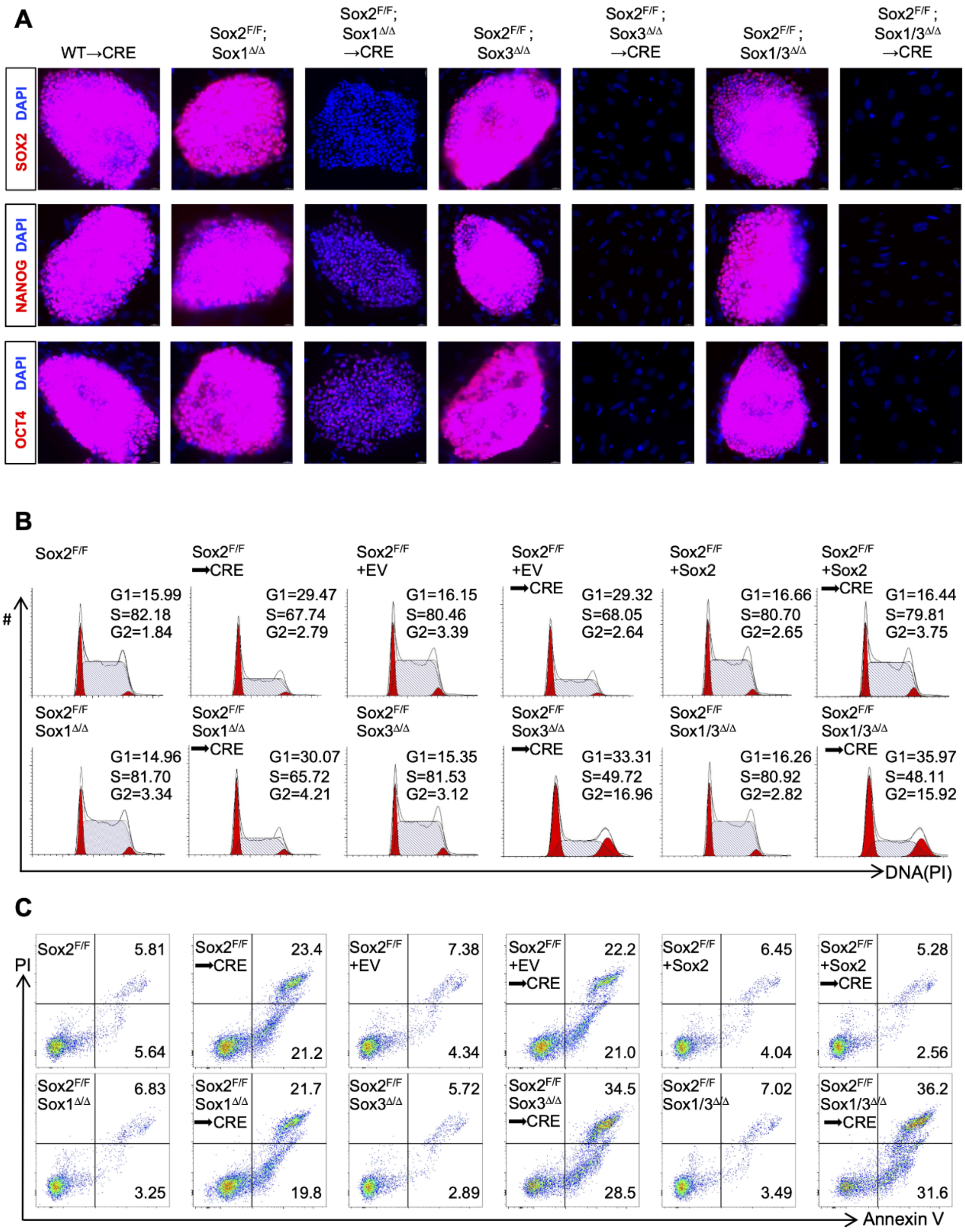
A
WT→CRE
Sox2F/F; Sox1Δ/Δ
Sox2F/F; Sox1Δ/Δ →CRE
Sox2F/F ; Sox3Δ/Δ
Sox2F/F ; Sox3Δ/Δ →CRE
Sox2F/F ; Sox1/3Δ/Δ
Sox2F/F ; Sox1/3Δ/Δ →CRE
SOX2 DAPI
NANOG DAPI
OCT4 DAPI
B
#
DNA(PI)
Sox2F/F
G1=15.99 S=82.18 G2=1.84
Sox2F/F →CRE
G1=29.47 S=67.74 G2=2.79
Sox2F/F +EV
G1=16.15 S=80.46 G2=3.39
Sox2F/F +EV →CRE
G1=29.32 S=68.05 G2=2.64
Sox2F/F +Sox2
G1=16.66 S=80.70 G2=2.65
Sox2F/F +Sox2 →CRE
G1=16.44 S=79.81 G2=3.75
Sox2F/F Sox1Δ/Δ
G1=14.96 S=81.70 G2=3.34
Sox2F/F Sox1Δ/Δ →CRE
G1=30.07 S=65.72 G2=4.21
Sox2F/F Sox3Δ/Δ
G1=15.35 S=81.53 G2=3.12
Sox2F/F Sox3Δ/Δ →CRE
G1=33.31 S=49.72 G2=16.96
Sox2F/F Sox1/3Δ/Δ
G1=16.26 S=80.92 G2=2.82
Sox2F/F Sox1/3Δ/Δ →CRE
G1=35.97 S=48.11 G2=15.92
C
PI
Annexin V
Sox2F/F 5.81 5.64
Sox2F/F →CRE 23.4 21.2
Sox2F/F +EV 7.38 4.34
Sox2F/F +EV →CRE 22.2 21.0
Sox2F/F +Sox2 6.45 4.04
Sox2F/F +Sox2 →CRE 5.28 2.56
Sox2F/F Sox1Δ/Δ 6.83 3.25
Sox2F/F Sox1Δ/Δ →CRE 21.7 19.8
Sox2F/F Sox3Δ/Δ 5.72 2.89
Sox2F/F Sox3Δ/Δ →CRE 34.5 28.5
Sox2F/F Sox1/3Δ/Δ 7.02 3.49
Sox2F/F Sox1/3Δ/Δ →CRE 36.2 31.6

Figure S3. The observed defects in ESCs deficient in Soxb1 family genes. A. Immunofluorescence analysis for OCT4, NANOG, SOX2 (red), or DAPI (blue) in ESC colonies of indicated genotypes. The images were captured using a confocal microscopy with a 63x objective lens. All images were shown merged with the blue and red fluorescent channel. B. Flow cytometric analysis of cell cycle with propidium iodide (PI) DNA staining. C. Flow cytometric analysis of cells staining with Annexin V FITC and PI. The percentages of Annexin V-positive (apoptotic) cells are within the two right quadrants. EV, empty vector.

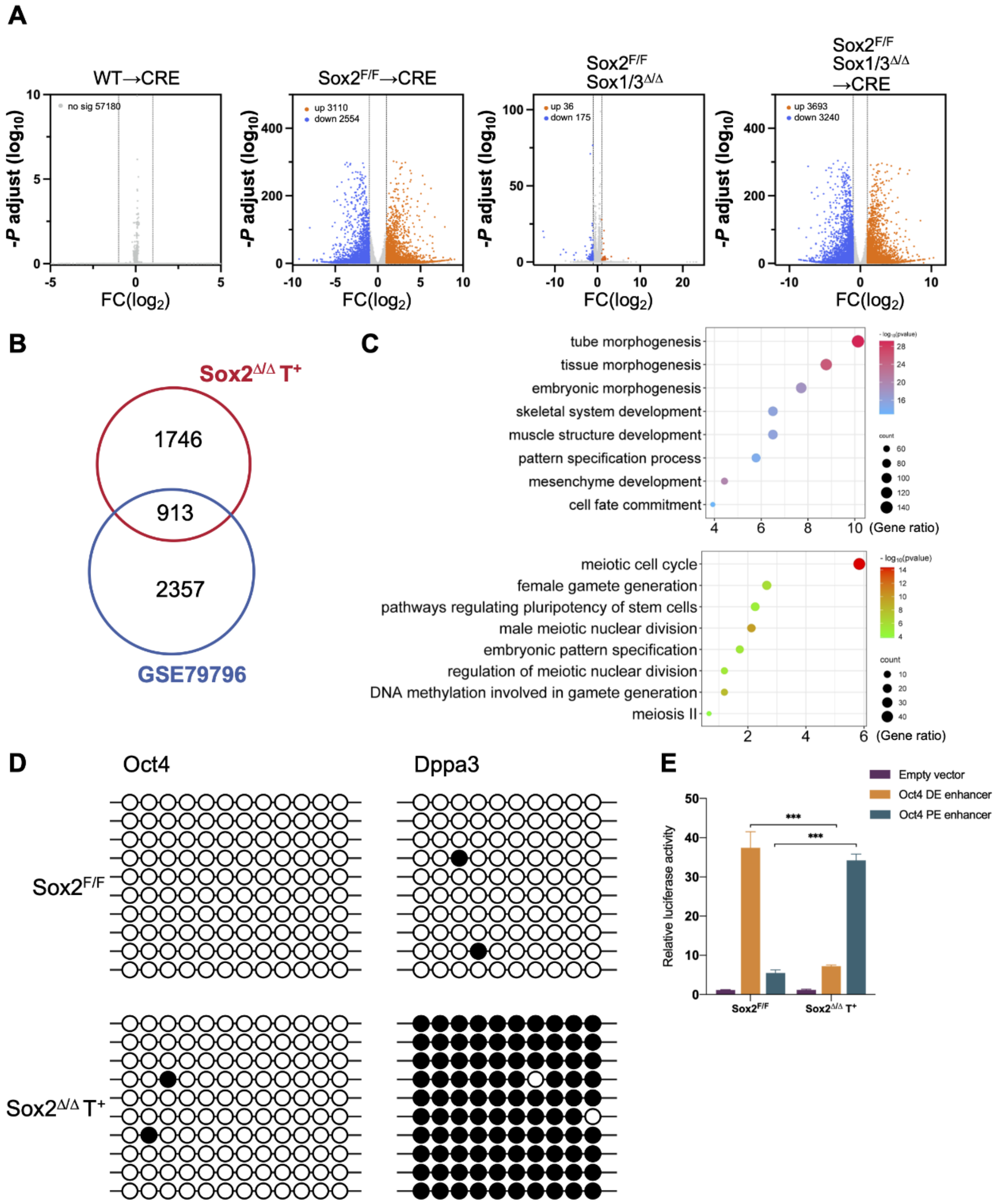
A
WT→CRE
no sig 57180
Sox2F/F→CRE
up 3110
down 2554
Sox2F/F Sox1/3Δ/Δ
up 36
down 175
Sox2F/F Sox1/3Δ/Δ →CRE
up 3693
down 3240
-P adjust (log10)
FC(log2)
B
Sox2Δ/Δ T+
1746
913
2357
GSE79796
C
tube morphogenesis
tissue morphogenesis
embryonic morphogenesis
skeletal system development
muscle structure development
pattern specification process
mesenchyme development
cell fate commitment
(Gene ratio)
meiotic cell cycle
female gamete generation
pathways regulating pluripotency of stem cells
male meiotic nuclear division
embryonic pattern specification
regulation of meiotic nuclear division
DNA methylation involved in gamete generation
meiosis II
D
Oct4
Dppa3
Sox2F/F
Sox2Δ/Δ T+
E
Empty vector
Oct4 DE enhancer
Oct4 PE enhancer
Relative luciferase activity
***
***
Sox2F/F
Sox2Δ/Δ T+

Figure S4. Sox2$^{\Delta/\Delta}$ T^{+} cells represent a primed pluripotent state. A. Volcano plots of $-\log_{10}$ (P value) against $\log_2$ fold change representing the differences in gene expression in ESCs of indicated genotypes. Upregulated (orange) and downregulated (blue) genes are highlighted. FC, fold change. B. Venn diagram showing the significant overlapping of differentially expressed genes between Sox2$^{\Delta/\Delta}$ T^{+} and EpiSCs. Published RNA-seq data were acquired from the NCBI (GEO: GSE79796). C. GO analysis of genes up-regulated (top) and down-regulated (bottom) in Sox2$^{\Delta/\Delta}$ T^{+} cells. D. Bisulfite sequencing profiles of DNA methylation of the Dppa3 locus. Black and white circles represent methylated and unmethylated CpGs, respectively. The numbers under the bisulfite sequencing profiles show the percentages of methylated CpG. E. Evaluation of Oct4 DE and PE reporter gene activity in Sox2$^{F/F}$ ESCs and Sox2$^{\Delta/\Delta}$ T^{+} cells. Data were obtained from three independent experiments and expressed as the mean ± SD. (*$p < 0.05$, **$p < 0.01$, ***$p < 0.001$).

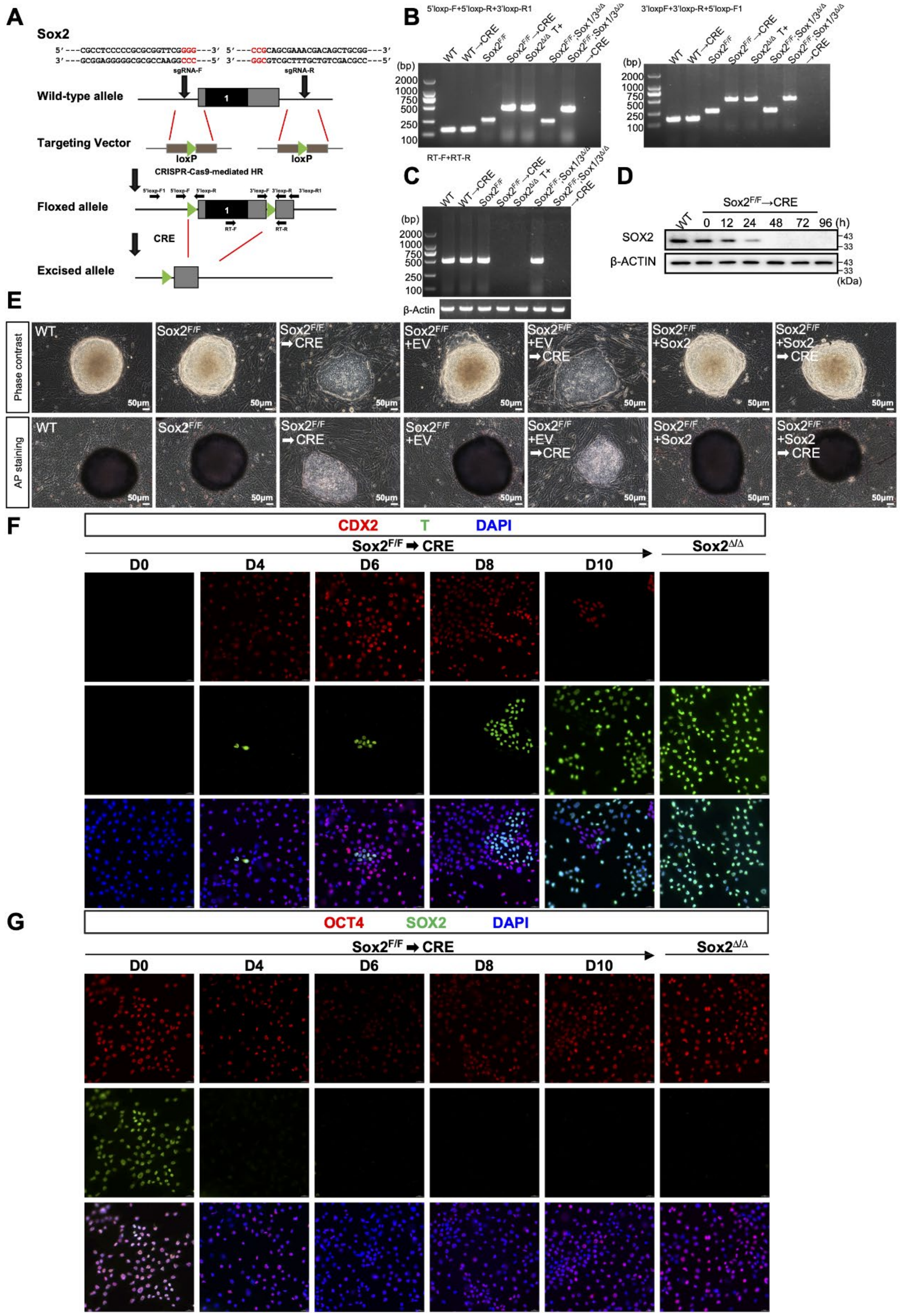
A
Sox2
sgRNA-F
sgRNA-R
Wild-type allele
Targeting Vector
loxP
loxP
CRISPR-Cas9-mediated HR
Floxed allele
CRE
Excised allele
B
5'loxp-F+5'loxp-R+3'loxp-R1
3'loxpF+3'loxp-R+5'loxp-F1
C
RT-F+RT-R
β-Actin
D
Sox2F/F→CRE
SOX2
β-ACTIN
(kDa)
E
Phase contrast
AP staining
F
CDX2 T DAPI
Sox2F/F ➡ CRE
D0 D4 D6 D8 D10
G
OCT4 SOX2 DAPI
Sox2F/F ➡ CRE
D0 D4 D6 D8 D10

Figure S5. Sox2-deficient E14Tg2a lines exist in a primed state of pluripotency. A. Schematic representation of CRISPR/Cas9 mediated knockout strategy to generate ESCs (E14Tg2a) deficient in Sox2. Left, PAM sequences are in red following the sgRNA sequence in black. The locations of genomic PCR primers and RT-PCR primers are shown. B. Genotyping of ESCs with Sox2 gene deletion using primers located upstream and downstream of the deleted region. C. Analysis of Sox2 gene expression in different samples as determined by RT-PCR. β-actin was used as an internal control. D. Western blot showing Sox2 levels in $Sox2^{F/F}$ infected by lenti-Cre virus at different time points. β-actin acted as a loading control. E. Representative phase contrast pictures (top) and AP staining images (bottom) of ESC colonies of indicated genotypes grown on a layer of MEFs. F and G. Immunofluorescence analysis for CDX2 (red), T (green) (F), OCT4 (red) and Sox2 (green) (G), or DAPI (blue) in $Sox2^{F/F}$, $Sox2^{\Delta/\Delta}$ or $Sox2^{F/F}$ ESCs following lenti-Cre infection. The images were captured using a confocal microscopy with a 63x objective lens. Merge, merged images. D, day.

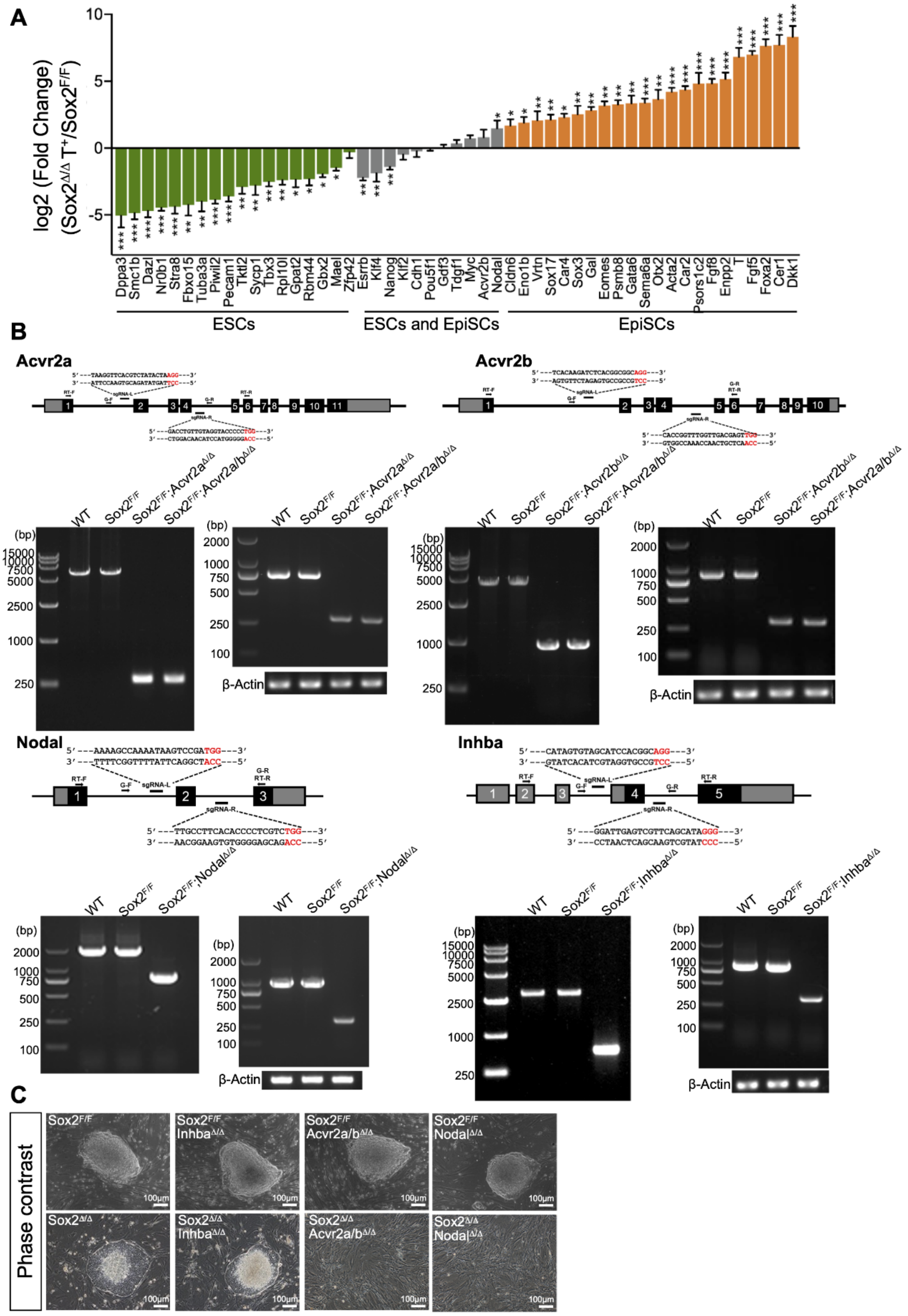
A
log2 (Fold Change)
(Sox2Δ/Δ T+/Sox2F/F)
10
5
0
-5
Dppa3
Smc1b
Dazl
Nr0b1
Stra8
Fbxo15
Tuba3a
Piwil2
Pecam1
Tktl2
Sycp1
Tbx3
Rpl10l
Gpat2
Rbm44
Gbx2
Mael
Zfp42
Esrrb
Klf4
Nanog
Klf2
Cdh1
Pou5f1
Gdf3
Tdgf1
Myc
Acvr2b
Nodal
Cldn6
Eno1b
Vrtn
Sox17
Car4
Sox3
Gal
Eomes
Psmb8
Gata6
Sema6a
Otx2
Acta2
Car2
Psors1c2
Fgf8
Enpp2
T
Fgf5
Foxa2
Cer1
Dkk1
ESCs
ESCs and EpiSCs
EpiSCs
B
Acvr2a
Acvr2b
WT
Sox2F/F
Sox2F/F;Acvr2aΔ/Δ
Sox2F/F;Acvr2a/bΔ/Δ
Sox2F/F;Acvr2bΔ/Δ
(bp)
β-Actin
Nodal
5′---AAAAGCCAAAATAAGTCCGATGG---3′
3′---TTTTCGGTTTTATTCAGGCTACC---5′
5′---TTGCCTTCACACCCCTCGTCTGG---3′
3′---AACGGAAGTGTGGGGAGCAGACC---5′
Sox2F/F;NodalΔ/Δ
Inhba
5′---CATAGTGTAGCATCCACGGCAGG---3′
3′---GTATCACATCGTAGGTGCCGTCC---5′
5′---GGATTGAGTCGTTCAGCATAGGG---3′
3′---CCTAACTCAGCAAGTCGTATCCC---5′
Sox2F/F;InhbaΔ/Δ
RT-F
RT-R
G-F
G-R
sgRNA-L
sgRNA-R
C
Phase contrast
Sox2F/F
Sox2F/F InhbaΔ/Δ
Sox2F/F Acvr2a/bΔ/Δ
Sox2F/F NodalΔ/Δ
Sox2Δ/Δ
Sox2Δ/Δ InhbaΔ/Δ
Sox2Δ/Δ Acvr2a/bΔ/Δ
Sox2Δ/Δ NodalΔ/Δ
100μm

Figure S6. Essential role of Nodal signaling pathway in the transition from naïve to primed state. A. RT–qPCR analysis of the expression of pluripotency markers specific for ESCs and/or EpiSCs in Sox2$^{\Delta/\Delta}$ T+ cells (129 strain, E14Tg2a). Data were obtained from three independent experiments and expressed as the mean ± SD. (*p < 0.05, **p < 0.01, ***p < 0.001). B. Top, schematic representation of CRISPR/Cas9 mediated knockout approaches to generate ESCs deficient in Acvr2a, Acvr2b, Nodal and Inhba. PAM sequences are in red following the sgRNA sequence. The locations of genomic PCR primers (G-F and G-R) and RT-PCR primers (RT-F and RT-R) are shown. Bottom, genotyping of ESCs with indicated gene deletions using primers located upstream and downstream of the deleted region (left). RT-PCR analysis for residual mRNA revealed a shorter band in the mutants (right). β-actin was used as an internal control. C. Phase-contrast images of colonies cultured on a layer of MEFs. Scale bar, 100 μm.

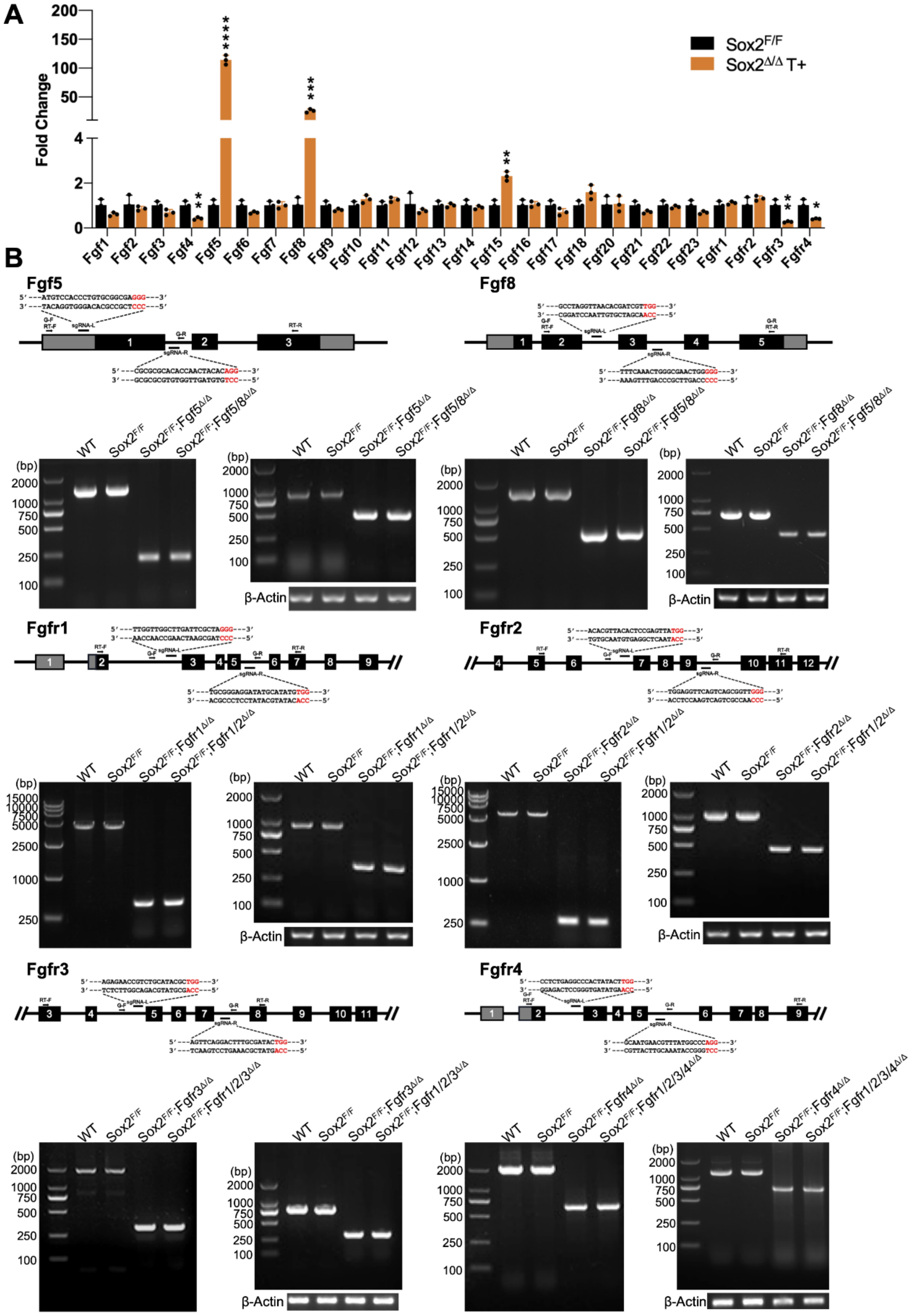
A
Fold Change
Sox2F/F
Sox2Δ/Δ T+
Fgf1
Fgf2
Fgf3
Fgf4
Fgf5
Fgf6
Fgf7
Fgf8
Fgf9
Fgf10
Fgf11
Fgf12
Fgf13
Fgf14
Fgf15
Fgf16
Fgf17
Fgf18
Fgf20
Fgf21
Fgf22
Fgf23
Fgfr1
Fgfr2
Fgfr3
Fgfr4
B
Fgf5
Fgf8
Fgfr1
Fgfr2
Fgfr3
Fgfr4
WT
Sox2F/F
Sox2F/F;Fgf5Δ/Δ
Sox2F/F;Fgf5/8Δ/Δ
Sox2F/F;Fgf8Δ/Δ
Sox2F/F;Fgfr1Δ/Δ
Sox2F/F;Fgfr1/2Δ/Δ
Sox2F/F;Fgfr2Δ/Δ
Sox2F/F;Fgfr3Δ/Δ
Sox2F/F;Fgfr1/2/3Δ/Δ
Sox2F/F;Fgfr4Δ/Δ
Sox2F/F;Fgfr1/2/3/4Δ/Δ
(bp)
β-Actin

Figure S7. Critical role for Fgf signaling controlling the exit from naïve pluripotency during the progression to primed pluripotency. A. RT-qPCR of Fgf family members and their receptors in $Sox2^{\Delta/\Delta}$ T^{+} cells. All data are normalized to β-actin and shown relative to control ESCs (set at 1.0). Data were obtained from three independent experiments and expressed as the mean ± SD. (*$p < 0.05$, **$p < 0.01$, ***$p < 0.001$). B. Top, schematic representation of CRISPR/Cas9 mediated knockout approaches to generate ESCs deficient in Fgf5, Fgf8, and Fgfr1-Fgfr4. PAM sequences are in red following the sgRNA sequence. The locations of genomic PCR primers (G-F and G-R) and RT-PCR primers (RT-F and RT-R) are shown. Bottom, genotyping of ESCs with indicated gene deletions using primers located upstream and downstream of the deleted region (left). RT-PCR analysis for residual mRNA revealed a shorter band in the mutants (right). β-actin was used as an internal control.

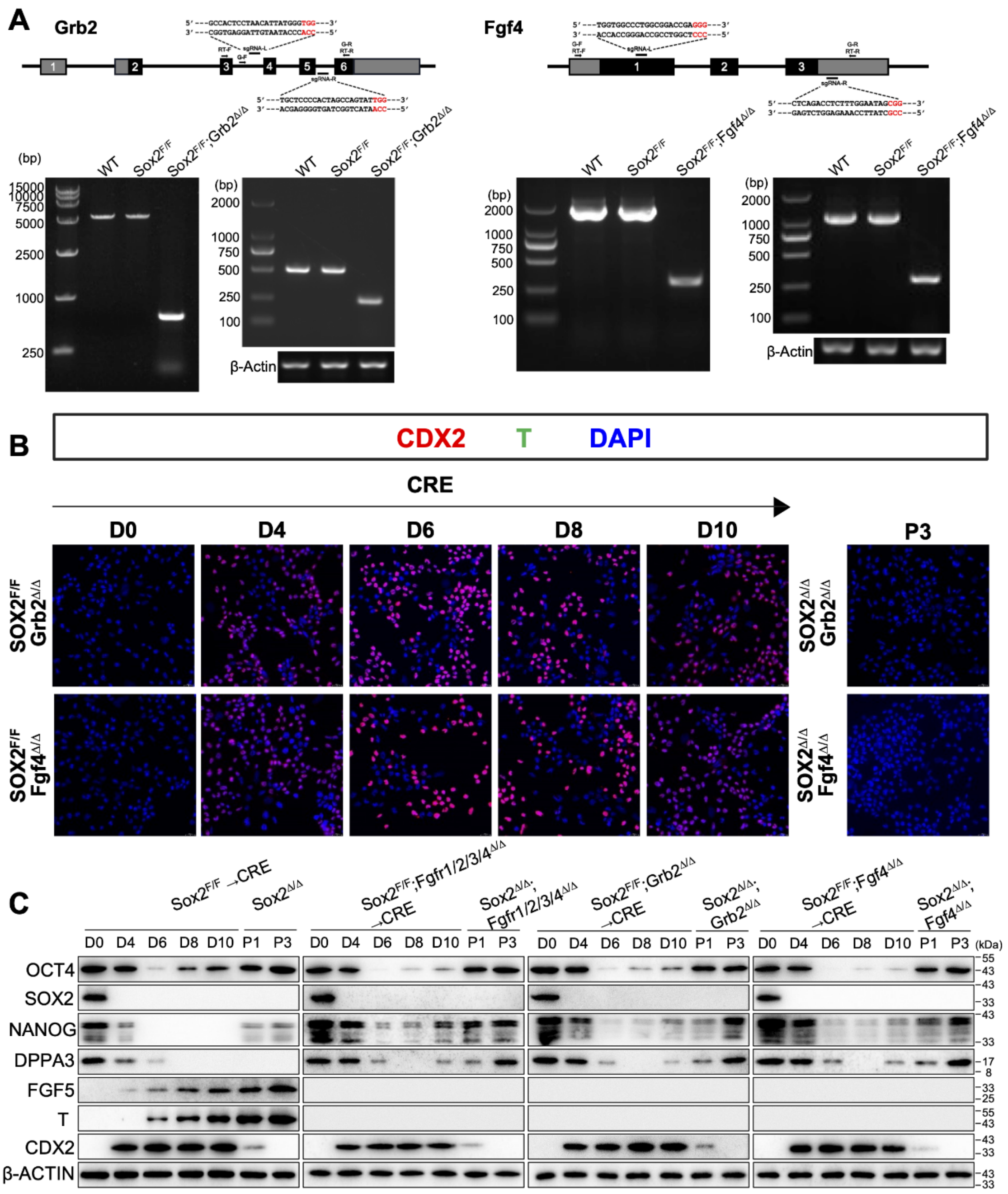
A
Grb2
Fgf4
WT
Sox2F/F
Sox2F/F;Grb2Δ/Δ
Sox2F/F;Fgf4Δ/Δ
(bp)
β-Actin
B
CDX2
T
DAPI
CRE
D0
D4
D6
D8
D10
P3
SOX2F/F Grb2Δ/Δ
SOX2F/F Fgf4Δ/Δ
SOX2Δ/Δ Grb2Δ/Δ
SOX2Δ/Δ Fgf4Δ/Δ
C
Sox2F/F →CRE
Sox2Δ/Δ
Sox2F/F;Fgfr1/2/3/4Δ/Δ →CRE
Sox2Δ/Δ; Fgfr1/2/3/4Δ/Δ
Sox2F/F;Grb2Δ/Δ →CRE
Sox2Δ/Δ; Grb2Δ/Δ
Sox2F/F;Fgf4Δ/Δ →CRE
Sox2Δ/Δ; Fgf4Δ/Δ
D0 D4 D6 D8 D10 P1 P3
OCT4
SOX2
NANOG
DPPA3
FGF5
T
CDX2
β-ACTIN
(kDa)

Figure S8. Fgf signaling is essential for the establishment of primed pluripotency in $Sox2^{\Delta/\Delta}$ T^{+} cells. A. Top, schematic representation of CRISPR/Cas9 mediated knockout approaches to generate ESCs deficient in Grb2 and Fgf4. PAM sequences are in red following the sgRNA sequence. The locations of genomic PCR primers (G-F and G-R) and RT-PCR primers (RT-F and RT-R) are shown. Bottom, genotyping of ESCs with indicated gene deletions using primers located upstream and downstream of the deleted region (left). RT-PCR analysis for residual mRNA revealed a shorter band in the mutants (right). β-actin was used as an internal control. B. Immunofluorescence analysis for CDX2 (red), T (green) or DAPI (blue) in ESCs of indicated genotypes following lenti-Cre infection. All images were shown merged with the green and red fluorescent channel. The images were captured using a confocal microscopy with a 63x objective lens. C. Western blot analysis of indicated protein levels in cells with indicated genotypes. β-Actin served as a loading control.

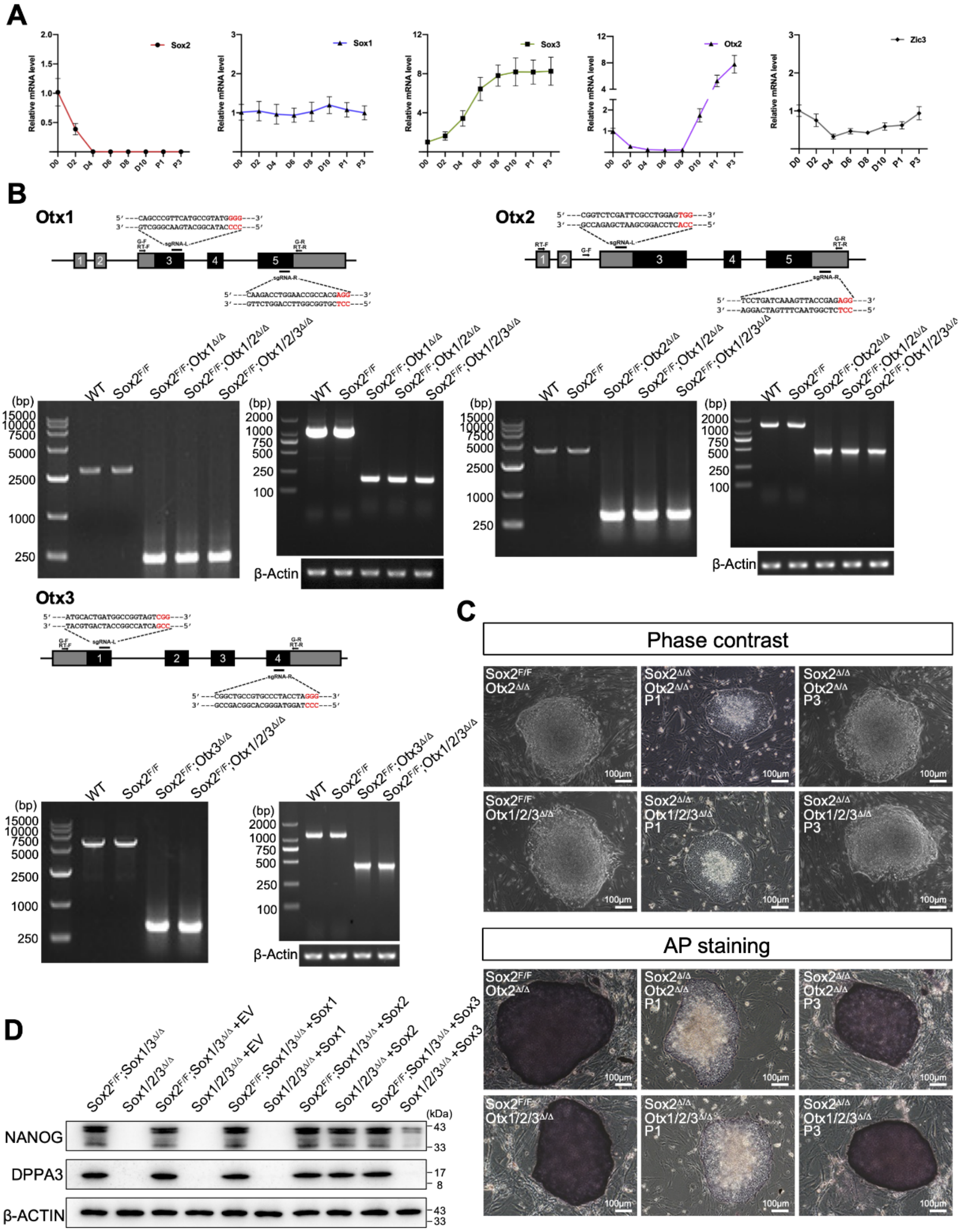
A
Relative mRNA level
Sox2
Sox1
Sox3
Otx2
Zic3
D0 D2 D4 D6 D8 D10 P1 P3
B
Otx1
Otx2
Otx3
sgRNA-L
sgRNA-R
G-F
RT-F
G-R
RT-R
WT
Sox2$^{F/F}$
Sox2$^{F/F}$;Otx1$^{Δ/Δ}$
Sox2$^{F/F}$;Otx1/2$^{Δ/Δ}$
Sox2$^{F/F}$;Otx1/2/3$^{Δ/Δ}$
Sox2$^{F/F}$;Otx2$^{Δ/Δ}$
Sox2$^{F/F}$;Otx3$^{Δ/Δ}$
(bp)
β-Actin
C
Phase contrast
AP staining
Sox2$^{F/F}$ Otx2$^{Δ/Δ}$
Sox2$^{Δ/Δ}$ Otx2$^{Δ/Δ}$ P1
Sox2$^{Δ/Δ}$ Otx2$^{Δ/Δ}$ P3
Sox2$^{F/F}$ Otx1/2/3$^{Δ/Δ}$
Sox2$^{Δ/Δ}$ Otx1/2/3$^{Δ/Δ}$ P1
Sox2$^{Δ/Δ}$ Otx1/2/3$^{Δ/Δ}$ P3
100μm
D
Sox2$^{F/F}$;Sox1/3$^{Δ/Δ}$
Sox1/2/3$^{Δ/Δ}$
Sox2$^{F/F}$;Sox1/3$^{Δ/Δ}$+EV
Sox1/2/3$^{Δ/Δ}$+EV
Sox2$^{F/F}$;Sox1/3$^{Δ/Δ}$+Sox1
Sox1/2/3$^{Δ/Δ}$+Sox1
Sox2$^{F/F}$;Sox1/3$^{Δ/Δ}$+Sox2
Sox1/2/3$^{Δ/Δ}$+Sox2
Sox2$^{F/F}$;Sox1/3$^{Δ/Δ}$+Sox3
Sox1/2/3$^{Δ/Δ}$+Sox3
(kDa)
NANOG
DPPA3
β-ACTIN
43
33
17
8

Figure S9. Functional compensation between Sox2 and Sox3 in maintaining the primed pluripotent state of Sox2$^{\Delta/\Delta}$ T^{+} cells. A. RT-qPCR analysis for selected transcripts in Sox2$^{F/F}$ ESCs after lenti-Cre infection. Relative expression is reflected as fold difference over uninfected ESCs normalized to β-actin. Data are expressed as means ± SEM of three biological replicates. D, day; P, passage. B. Top, schematic representation of CRISPR/Cas9 mediated knockout approaches to generate ESCs deficient in Otx family. PAM sequences are in red following the sgRNA sequence. The locations of genomic PCR primers (G-F and G-R) and RT-PCR primers (RT-F and RT-R) are shown. Bottom, genotyping of ESCs with indicated gene deletions using primers located upstream and downstream of the deleted region (left); RT-PCR analysis for residual mRNA revealed a shorter band in the mutants (right). β-actin was used as an internal control. C. Top, phase-contrast images of colonies cultured on a layer of MEFs. Bottom, representative images of AP staining of colonies of indicated genotypes cultured on a feeder layer of MEFs. Scale bar, 100 μm. D. Western blot analysis of indicated protein levels in cells with indicated genotypes. β-Actin served as a loading control.

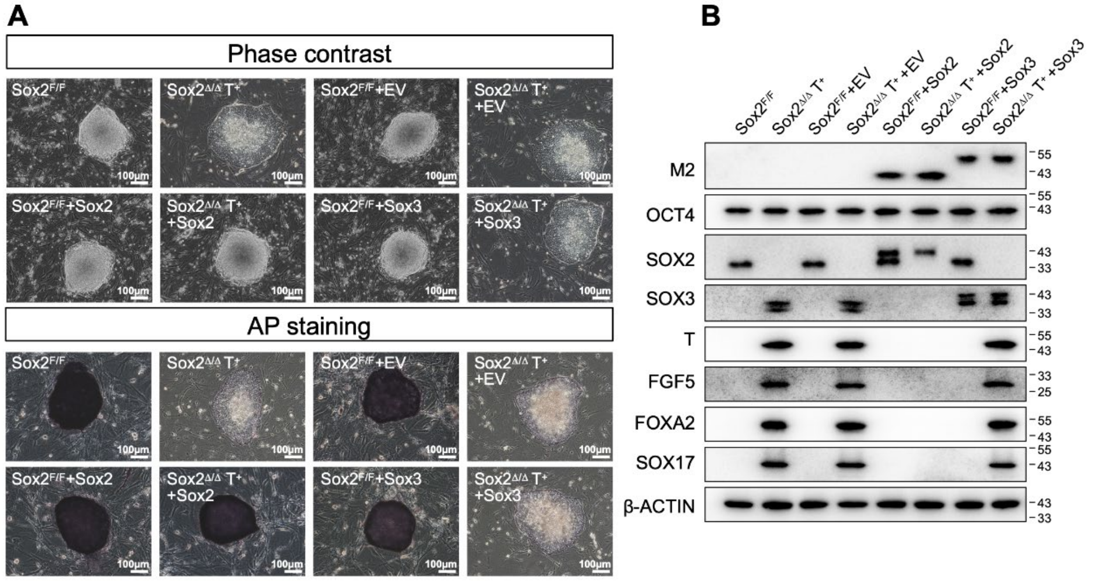

Figure S10. Sox3 compensates for the absence of Sox2 in the maintenance of primed pluripotency. A. Top, phase-contrast images of colonies cultured on a layer of MEFs. Bottom, Representative images of AP staining of colonies of indicated genotypes cultured on a feeder layer of MEFs. Scale bar, 100 μm. B. Western blot for selected proteins in ESCs of the indicated genotypes. β-Actin served as a loading control. EV, empty vector.

Supplementary Table

Table S1. Genes that are differentially expressed in $Sox2^{F/F}$-Cre vs. $Sox2^{F/F}$ ESCs

Table S2. Genes that are differentially expressed in $Oct4^{\Delta/\Delta}$ vs. $Oct4^{F/F}$ ESCs

Table S3. Genes that are differentially expressed in $Nanog^{\Delta/\Delta}$ vs. $Nanog^{F/F}$ ESCs

Table S4. Genes that are differentially expressed in $Sox2^{F/F}$;$Sox1/3^{\Delta/\Delta}$ vs. $Sox2^{F/F}$ ESCs

Table S5. Genes that are differentially expressed in $Sox1/2/3^{\Delta/\Delta}$ vs. $Sox2^{F/F}$ ESCs

Table S6. Genes that are differentially expressed in $Sox2^{\Delta/\Delta}$ vs. $Sox2^{F/F}$ ESCs

Table S7. Summary of the primary antibodies and oligonucleotides used in the present study
