# Supplementary material for: Loss of sex-determining region Y-box 2 (Sox2) captures embryonic stem cells in a primed pluripotent state
Source: J Biol Chem. 2025 Apr 9;301(5):108501. doi: 10.1016/j.jbc.2025.108501 (PMC12135381; doi:10.1016/j.jbc.2025.108501)
Supplement: Table S7 [file mmc8.docx]

**Table S7.** Summary of the primary antibodies and oligonucleotides used in the present study

| REAGENT or RESOURCE | SOURCE | | | IDENTIFIER |  |
| --- | --- | --- | --- | --- | --- |
| Antibodies | | | | |  |
| Rabbit anti-Oct4 | Cell Signaling Technology | | | Cat# 83932 |  |
| Mouse anti-Sox2 | Cell Signaling Technology | | | Cat# 4900 |  |
| Rabbit anti-Nanog | Abcam | | | Cat# ab214549 |  |
| Rabbit anti-T | Abcam | | | Cat# ab209665 |  |
| Rabbit anti-Foxa2 | Abcam | | | Cat# ab108422 |  |
| Rabbit anti-Sox17 | Abcam | | | Cat# ab224637 |  |
| Mouse anti-Stella | Santa Cruz Biotechnology | | | Cat# sc-376862 |  |
| Rabbit anti-Cdx2 | Cell Signaling Technology | | | Cat# 12306 |  |
| Rabbit anti-Fgf5 | Abcam | | | Cat# ab88118 |  |
| Mouse anti-βActin | Proteintech | | | Cat# 60008-1-Ig |  |
| Rabbit anti-Flag | Sigma-Aldrich | | | Cat# F1804 |  |
| Rabbit anti-Sox3 | Abcam | | | Cat# ab183606 |  |
| Goat anti-T | ThermoFisher | | | Cat# PA5-46984 |  |
| Mouse anti-Nanog | Abcam | | | Cat# ab173368 |  |
| Oligonucleotides | | | | |  |
| Sox1 upstream sgRNA:  GCCGCGGCGCGCTCATTTAA | | GeneScript | sgRNA | | |
| Sox1 downstream sgRNA:  TTTAGGCGTTGCGATACGGG | | GeneScript | sgRNA | | |
| Sox2 upstream sgRNA:  CGCCTCCCCCGCGCGGTTCG | | GeneScript | sgRNA | | |
| Sox2 downstream sgRNA:  CCGCAGCTGTCGTTTCGCTG | | GeneScript | sgRNA | | |
| Sox3 upstream sgRNA:  AAGTTGTTTCCGAACCCCGC | | GeneScript | sgRNA | | |
| Sox3 downstream sgRNA:  TCCACCGCGGTAGCTAACGC | | GeneScript | sgRNA | | |
| Nanog upstream sgRNA:  ACCCCACCCCGTGAACTGAC | | GeneScript | sgRNA | | |
| Nanog downstream sgRNA:  GGACAAGCAAGCACCTTAAT | | GeneScript | sgRNA | | |
| Oct4 upstream sgRNA:  GGATTACATGCTCGTTCGTC | | GeneScript | sgRNA | | |
| Oct4 downstream sgRNA:  TACTGGCTTTCTCCAACCGC | | GeneScript | sgRNA | | |
| Fgf4 upstream sgRNA:  TGGTGGCCCTGGCGGACCGA | | GeneScript | sgRNA | | |
| Fgf4 downstream sgRNA:  CTCAGACCTCTTTGGAATAG | | GeneScript | sgRNA | | |
| Fgf5 upstream sgRNA:  ATGTCCACCCTGTGCGGCGA | | GeneScript | sgRNA | | |
| Fgf5 downstream sgRNA:  CGCGCGCACACCAACTACAC | | GeneScript | sgRNA | | |
| Fgf8 upstream sgRNA:  GCCTAGGTTAACACGATCGT | | GeneScript | sgRNA | | |
| Fgf8 downstream sgRNA:  TTTCAAACTGGGCGAACTGG | | GeneScript | sgRNA | | |
| Fgfr1 upstream sgRNA:  TTGGTTGGCTTGATTCGCTA | | GeneScript | sgRNA | | |
| Fgfr1 downstream sgRNA:  TGCGGGAGGATATGCATATG | | GeneScript | sgRNA | | |
| Fgfr2 upstream sgRNA:  ACACGTTACACTCCGAGTTA | | GeneScript | sgRNA | | |
| Fgfr2 downstream sgRNA:  TGGAGGTTCAGTCAGCGGTT | | GeneScript | sgRNA | | |
| Fgfr3 upstream sgRNA:  AGAGAACCGTCTGCATACGC | | GeneScript | sgRNA | | |
| Fgfr3 downstream sgRNA:  AGTTCAGGACTTTGCGATAC | | GeneScript | sgRNA | | |
| Fgfr4 upstream sgRNA:  CCTCTGAGGCCCACTATACT | | GeneScript | sgRNA | | |
| Fgfr4 downstream sgRNA:  GCAATGAACGTTTATGGCCC | | GeneScript | sgRNA | | |
| Inhba upstream sgRNA:  CATAGTGTAGCATCCACGGC | | GeneScript | sgRNA | | |
| Inhba downstream sgRNA:  GGATTGAGTCGTTCAGCATA | | GeneScript | sgRNA | | |
| Acvr2a upstream sgRNA:  TAAGGTTCACGTCTATACTA | | GeneScript | sgRNA | | |
| Acvr2a downstream sgRNA:  GACCTGTTGTAGGTACCCCC | | GeneScript | sgRNA | | |
| Acvr2b upstream sgRNA:  TCACAAGATCTCACGGCGGC | | GeneScript | sgRNA | | |
| Acvr2b downstream sgRNA:  CACCGGTTTGGTTGACGAGT | | GeneScript | sgRNA | | |
| Nodal upstream sgRNA:  AAAAGCCAAAATAAGTCCGA | | GeneScript | sgRNA | | |
| Nodal downstream sgRNA:  TTGCCTTCACACCCCTCGTC | | GeneScript | sgRNA | | |
| Otx1 upstream sgRNA:  CAGCCCGTTCATGCCGTATG | | GeneScript | sgRNA | | |
| Otx1 downstream sgRNA:  CAAGACCTGGAACCGCCACG | | GeneScript | sgRNA | | |
| Otx2 upstream sgRNA:  CGGTCTCGATTCGCCTGGAG | | GeneScript | sgRNA | | |
| Otx2 downstream sgRNA:  TCCTGATCAAAGTTACCGAG | | GeneScript | sgRNA | | |
| Otx3 upstream sgRNA:  ATGCACTGATGGCCGGTAGT | | GeneScript | sgRNA | | |
| Otx3 downstream sgRNA:  CGGCTGCCGTGCCCTACCTA | | GeneScript | sgRNA | | |
| Grb2 upstream sgRNA:  GCCACTCCTAACATTATGGG | | GeneScript | sgRNA | | |
| Grb2 downstream sgRNA:  TGCTCCCCACTAGCCAGTAT | | GeneScript | sgRNA | | |
| Dnmt1 upstream sgRNA:  CCTTGGGTTTCCGTTTAGTG | | GeneScript | sgRNA | | |
| Dnmt1 downstream sgRNA:  GGGCTCCTTTGTGAGCCGAG | | GeneScript | sgRNA | | |
| Dnmt3a upstream sgRNA:  AGCTCACAGTGGCGTCTGCG | | GeneScript | sgRNA | | |
| Dnmt3a downstream sgRNA:  CTGCCTGAGACTAGACACAT | | GeneScript | sgRNA | | |
| Dnmt3b upstream sgRNA:  TTCACACCGAGTTTACGTGT | | GeneScript | sgRNA | | |
| Dnmt3b downstream sgRNA:  ATTACCTTCCCATCTTTAAG | | GeneScript | sgRNA | | |
| Dnmt3l upstream sgRNA:  TTAGTGGGCACTCCGGAGCT | | GeneScript | sgRNA | | |
| Dnmt3l downstream sgRNA:  GGTGCTGAAGGATGCGCATC | | GeneScript | sgRNA | | |
| Dppa2 upstream sgRNA:  TTGATAAGGCGCTATACAAA | | GeneScript | sgRNA | | |
| Dppa2 downstream sgRNA:  AAAAATTGAGGTTAAAGGAC | | GeneScript | sgRNA | | |
| Dppa3 upstream sgRNA:  CTGTCCAGAAGCATTTGAGC | | GeneScript | sgRNA | | |
| Dppa3 downstream sgRNA:  AGGCTATCCTCTAAGCAGTC | | GeneScript | sgRNA | | |
| Dppa5a upstream sgRNA:  CTCATTGCCTTTCCCCGGAC | | GeneScript | sgRNA | | |
| Dppa5a downstream sgRNA:  GCATCCAGGTCGGAGACACA | | GeneScript | sgRNA | | |
| Esrrb upstream sgRNA:  CTCCGTCCAGTTAGAAAACT | | GeneScript | sgRNA | | |
| Esrrb downstream sgRNA:  ATGAAAGAGCTGAATACCGC | | GeneScript | sgRNA | | |
| Fbxo15 upstream sgRNA:  GGTATTTGTAGACTCAGTAC | | GeneScript | sgRNA | | |
| Fbxo15 downstream sgRNA:  CATGATGTGTTCTTTGCCAC | | GeneScript | sgRNA | | |
| Foxd3 upstream sgRNA:  CCTCGCTCTCTGCTCGCTCC | | GeneScript | sgRNA | | |
| Foxd3 downstream sgRNA:  CAAACTCAACCCGCGTCCGC | | GeneScript | sgRNA | | |
| Gbx2 upstream sgRNA:  TGGTACTCCCAGCGTCCGGA | | GeneScript | sgRNA | | |
| Gbx2 downstream sgRNA:  CACACACGCTGTGATTATTG | | GeneScript | sgRNA | | |
| Gdf3 upstream sgRNA:  CGTGCGCTCGTGGGTGTGAT | | GeneScript | sgRNA | | |
| Gdf3 downstream sgRNA:  GATATAATATGGTACAGGGC | | GeneScript | sgRNA | | |
| Klf2 upstream sgRNA:  CTGAGGACACGCGCGCTGAA | | GeneScript | sgRNA | | |
| Klf2 downstream sgRNA:  GACCTGTGTGCTTTCGGTAG | | GeneScript | sgRNA | | |
| Klf4 upstream sgRNA:  ACCGTCGCCGCCAGGTCGTA | | GeneScript | sgRNA | | |
| Klf4 downstream sgRNA:  GGGTAGTTGGGCCCCGGATG | | GeneScript | sgRNA | | |
| Klf5 upstream sgRNA:  GAAGACATGTCGTAGAGTCT | | GeneScript | sgRNA | | |
| Klf5 downstream sgRNA:  TTACACGTTCAAACTAACCT | | GeneScript | sgRNA | | |
| Klf6 upstream sgRNA:  GCTTTCCTCTAGAATTCTCG | | GeneScript | sgRNA | | |
| Klf6 downstream sgRNA:  CCCAACATAGCACGATGCAT | | GeneScript | sgRNA | | |
| Klf15 upstream sgRNA:  CAGGCCCGTTGCACAATCCA | | GeneScript | sgRNA | | |
| Klf15 downstream sgRNA:  TGACTGGCTAAGAACCACCC | | GeneScript | sgRNA | | |
| Klf17 upstream sgRNA:  CAAAGATCCAAATACGACAA | | GeneScript | sgRNA | | |
| Klf17 downstream sgRNA:  TGACATCCAGAGCTTCGTGT | | GeneScript | sgRNA | | |
| Lefty1 upstream sgRNA:  GCTAACTCTTGCACACTTAG | | GeneScript | sgRNA | | |
| Lefty1 downstream sgRNA:  CAGCAGTGCGCGTCAGGCGA | | GeneScript | sgRNA | | |
| Lefty2 upstream sgRNA:  TAGAAGCCGGGTCATGGATC | | GeneScript | sgRNA | | |
| Lefty2 downstream sgRNA:  TCAAAGACTTACTCTTGAAG | | GeneScript | sgRNA | | |
| Nr0b1 upstream sgRNA:  CGGAGAAGAGCACCCGAGGC | | GeneScript | sgRNA | | |
| Nr0b1 downstream sgRNA:  GAACCAGTAAGCTTTGTACC | | GeneScript | sgRNA | | |
| Pecam1 upstream sgRNA:  GTTTGTTGTGCCAAAACGCT | | GeneScript | sgRNA | | |
| Pecam1 downstream sgRNA:  TATTGAGTATCCGGGCTTT | | GeneScript | sgRNA | | |
| Pou3f1 upstream sgRNA:  CTTCTGCACTTCGCGGTACG | | GeneScript | sgRNA | | |
| Pou3f1 downstream sgRNA:  TTCGCCGCTCGCTGGAGAGA | | GeneScript | sgRNA | | |
| Prdm14 upstream sgRNA:  AGCCAAGTGGTTTCCCTCGG | | GeneScript | sgRNA | | |
| Prdm14 downstream sgRNA:  ACCCACTTGTCAGAATAACC | | GeneScript | sgRNA | | |
| Sall4 upstream sgRNA:  GTGACTCTGCAGGCGCTCCG | | GeneScript | sgRNA | | |
| Sall4 downstream sgRNA:  AGCAAAGGCGGCATACCCGA | | GeneScript | sgRNA | | |
| Stat3 upstream sgRNA:  TATTAACACCTACTTGTCAT | | GeneScript | sgRNA | | |
| Stat3 downstream sgRNA:  GCTGGACACTATTGCTAAGA | | GeneScript | sgRNA | | |
| Tbx3 upstream sgRNA:  ATCCGCACCTTTATTAACCC | | GeneScript | sgRNA | | |
| Tbx3 downstream sgRNA:  CCTAATAAAAAGTTGTGAAT | | GeneScript | sgRNA | | |
| Tcl1 upstream sgRNA:  CCAGTGCGGTAGACATTGCT | | GeneScript | sgRNA | | |
| Tcl1 downstream sgRNA:  TAGCCAAACCCCTTGGCACC | | GeneScript | sgRNA | | |
| Tdgf1 upstream sgRNA:  TGTTACTGGAGACAGCGCGG | | GeneScript | sgRNA | | |
| Tdgf1 downstream sgRNA:  TCTGTAGGTGACGGGACAGT | | GeneScript | sgRNA | | |
| Tet1 upstream sgRNA:  CCTTTCACTGACTACGATGC | | GeneScript | sgRNA | | |
| Tet1 downstream sgRNA:  AGATTTCGTGGGGGTTACGG | | GeneScript | sgRNA | | |
| Tet2 upstream sgRNA:  TGCTTGCTAGATCTAGGTAT | | GeneScript | sgRNA | | |
| Tet2 downstream sgRNA:  CACTGTGGCCATGATTTGGC | | GeneScript | sgRNA | | |
| Tet3 upstream sgRNA:  TATAACACACCGTTCCGTGT | | GeneScript | sgRNA | | |
| Tet3 downstream sgRNA:  ACCCCTGCTGGAGGTCCCGT | | GeneScript | sgRNA | | |
| Tfcp2l1 upstream sgRNA:  TATCTATCTAGTGGGCGAGT | | GeneScript | sgRNA | | |
| Tfcp2l1 downstream sgRNA:  GGGACTAGGAGTCGGGCGCA | | GeneScript | sgRNA | | |
| Tfe3 upstream sgRNA:  GAACGTTGCCAAGCCTAATC | | GeneScript | sgRNA | | |
| Tfe3 downstream sgRNA:  CCTCCCCTAGACATCCCAAT | | GeneScript | sgRNA | | |
| Utf1 upstream sgRNA:  GGTGCGTGCACTCCAAGGCG | | GeneScript | sgRNA | | |
| Utf1 downstream sgRNA:  CTTTGCTAAGTCCGTTGGAG | | GeneScript | sgRNA | | |
| Zfp42 upstream sgRNA:  GATGTTGGCTTGCCGTAAAT | | GeneScript | sgRNA | | |
| Zfp42 downstream sgRNA:  TTCAATAGCACATATAGTAA | | GeneScript | sgRNA | | |
| Zic2 upstream sgRNA:  TTCGGAGTAAATATTGACGA | | GeneScript | sgRNA | | |
| Zic2 downstream sgRNA:  ATAAGCCATTAAAATCACCG | | GeneScript | sgRNA | | |
| Zic3 upstream sgRNA:  GAGACCCCCTAGGCGCTACC | | GeneScript | sgRNA | | |
| Zic3 downstream sgRNA:  GTAAAAGTGTGTGTAGTCCC | | GeneScript | sgRNA | | |
| Sox1 forward primer:  CCCCGACAGCGATTCTTTCT | | GeneScript | Genomic-PCR | | |
| Sox1 reverse primer:  TTTCACAGGGAAACGGGCTT | | GeneScript | Genomic-PCR | | |
| Sox2 5’ loxP forward primer1:  TGACACGGAAGCCAGGAG | | GeneScript | Genomic-PCR | | |
| Sox2 5’ loxP forward primer:  CCGTGACCTGTTGCTGAAAA | | GeneScript | Genomic-PCR | | |
| Sox2 5’ loxP reverse primer:  AGGGTTTTGCATGAAAGGGG | | GeneScript | Genomic-PCR | | |
| Sox2 3’ loxP forward primer:  GAATTGGGAGGGGTGCAAAA | | GeneScript | Genomic-PCR | | |
| Sox2 3’ loxP reverse primer:  TCTAGTCGGCATCACGGTTT | | GeneScript | Genomic-PCR | | |
| Sox2 3’ loxP reverse primer1:  TCAAAACCCAGCAAGAACCC | | GeneScript | Genomic-PCR | | |
| Sox3 forward primer:  ACACTCAACGTCCCAAGCAT | | GeneScript | Genomic-PCR | | |
| Sox3 reverse primer:  CCATACGACTCGAAGGACCC | | GeneScript | Genomic-PCR | | |
| Nanog 5’ loxP forward primer 1:  ACCCAGGGGTGACAAAGTAT | | GeneScript | Genomic-PCR | | |
| Nanog 5’ loxP forward primer:  AACTGACTGGCCTTTTTAAA | | GeneScript | Genomic-PCR | | |
| Nanog 5’ loxP reverse primer:  TGGTACATACCTTTAGTCCCA | | GeneScript | Genomic-PCR | | |
| Nanog 3’ loxP forward primer:  ACCAGGAGTTTGAGGGTAGC | | GeneScript | Genomic-PCR | | |
| Nanog 3’ loxP reverse primer:  ACATAGCAGTTACTCTTGGGG | | GeneScript | Genomic-PCR | | |
| Nanog 3’ loxP reverse primer 1:  CTCCCAACCGGTTAAGCCAA | | GeneScript | Genomic-PCR | | |
| Oct4 5’ loxP forward primer1:  CTACTACAGAAGGTTGTGAG | | GeneScript | Genomic-PCR | | |
| Oct4 5’ loxP forward primer:  ACTGCTGAGCCATGTGTTTC | | GeneScript | Genomic-PCR | | |
| Oct4 5’ loxP reverse primer:  CCCCAGAACCTAGCCATCTA | | GeneScript | Genomic-PCR | | |
| Oct4 3’ loxP forward primer:  CTGGCCTGTCTGTCACTCAT | | GeneScript | Genomic-PCR | | |
| Oct4 3’ loxP reverse primer:  TTCAATGGCGCCTGTTTTGA | | GeneScript | Genomic-PCR | | |
| Oct4 3’ loxP reverse primer1:  GCCCTCTTCTGGTGTGTCTA | | GeneScript | Genomic-PCR | | |
| Fgf4 forward primer:  TTGCGTCCCTATTTGCTCTC | | GeneScript | Genomic-PCR | | |
| Fgf4 reverse primer:  GGTCCTTCTCACTGCCTCAG | | GeneScript | Genomic-PCR | | |
| Fgf5 forward primer:  GTGCACGGAGCAGTGAGAT | | GeneScript | Genomic-PCR | | |
| Fgf5 reverse primer:  TCCGGTTTTCCAGATCATGT | | GeneScript | Genomic-PCR | | |
| Fgf8 forward primer:  AATCCAGCCCCAAACTACCC | | GeneScript | Genomic-PCR | | |
| Fgf8 reverse primer:  AGTCTGGAAATTGGCCCCAT | | GeneScript | Genomic-PCR | | |
| Fgfr1 forward primer:  ACCTCTTCCTTGGCTGTCCT | | GeneScript | Genomic-PCR | | |
| Fgfr1 reverse primer:  CAGCCTGGTCTGCATAGTGA | | GeneScript | Genomic-PCR | | |
| Fgfr2 forward primer:  TGAATCTGGGCATCATCTCA | | GeneScript | Genomic-PCR | | |
| Fgfr2 reverse primer:  ACTTGAACCCGAACATCTGG | | GeneScript | Genomic-PCR | | |
| Fgfr3 forward primer:  TTTCCCAGGCTGCATCTAGT | | GeneScript | Genomic-PCR | | |
| Fgfr3 reverse primer:  ACAGTGTGAGACGCATCAGA | | GeneScript | Genomic-PCR | | |
| Fgfr4 forward primer:  GGAAGGAAATGTGGCTGCTC | | GeneScript | Genomic-PCR | | |
| Fgfr4 reverse primer:  AGTGGAGGCAGGTTAGTTCC | | GeneScript | Genomic-PCR | | |
| Inhba forward primer:  ACAACAGCAGAGTCCTTGGT | | GeneScript | Genomic-PCR | | |
| Inhba reverse primer:  AGGTTAGTGTGGTCCTGAGC | | GeneScript | Genomic-PCR | | |
| Acvr2a forward primer:  TAGGACAACATACCAGCCGT | | GeneScript | Genomic-PCR | | |
| Acvr2a reverse primer:  TGAGGTGTCCAGAGTGAGAT | | GeneScript | Genomic-PCR | | |
| Acvr2b forward primer:  CACAAGTGTCAGGGCCTAGG | | GeneScript | Genomic-PCR | | |
| Acvr2b reverse primer:  CATGCAGGTATGAGAGGCCT | | GeneScript | Genomic-PCR | | |
| Nodal forward primer:  TAATCCCAGCACTTGGGAAG | | GeneScript | Genomic-PCR | | |
| Nodal reverse primer:  ACCCACACTCCTCCACAATC | | GeneScript | Genomic-PCR | | |
| Otx1 forward primer:  ACAGCTTGAAGGACTTCGCA | | GeneScript | Genomic-PCR | | |
| Otx1 reverse primer:  ATGGGGACCATGGAGGGTAT | | GeneScript | Genomic-PCR | | |
| Otx2 forward primer:  TGGTCTCACTCCATCCCCTC | | GeneScript | Genomic-PCR | | |
| Otx2 reverse primer:  ACCGGATCACCTCTGCTTTG | | GeneScript | Genomic-PCR | | |
| Otx3 forward primer:  AGCCCTTGGTAAAGTGGACG | | GeneScript | Genomic-PCR | | |
| Otx3 reverse primer:  GCCAGACAGTCAATTGGGGA | | GeneScript | Genomic-PCR | | |
| Grb2 forward primer:  CATACACGAGGCCTGAGGTT | | GeneScript | Genomic-PCR | | |
| Grb2 reverse primer:  GACATAATTGCGGGGAAACA | | GeneScript | Genomic-PCR | | |
| Tbx3 forward primer:  CTAACAGGCACACAGTTCGC | | GeneScript | Genomic-PCR | | |
| Tbx3 reverse primer:  GAGCCATTCACGTCTTCCTG | | GeneScript | Genomic-PCR | | |
| Tfe3 forward primer:  TCCTCGTACCTGACAAACCC | | GeneScript | Genomic-PCR | | |
| Tfe3 reverse primer:  GTAGCGTGTAGGGTTCTCGA | | GeneScript | Genomic-PCR | | |
| Tcl1 forward primer:  AGGCTGGCTAAGGTTGGTAA | | GeneScript | Genomic-PCR | | |
| Tcl1 reverse primer:  GGCCCAGCAGTCATCTCTTA | | GeneScript | Genomic-PCR | | |
| Zic3 forward primer:  TGCCAATCATTGTGTCGGTG | | GeneScript | Genomic-PCR | | |
| Zic3 reverse primer:  CTCGGGTGTGTGTAGGACTT | | GeneScript | Genomic-PCR | | |
| Zic2 forward primer:  ATGAATCTGGTGTGAGCCGA | | GeneScript | Genomic-PCR | | |
| Zic2 reverse primer:  CTCAAGCTGACTCCTTCCCA | | GeneScript | Genomic-PCR | | |
| Utf1 forward primer:  GCTAGACGATGGGAGGGTC | | GeneScript | Genomic-PCR | | |
| Utf1 reverse primer:  TCTCTCACAGAAGGGATCGC | | GeneScript | Genomic-PCR | | |
| Tdgf1 forward primer:  GAACTCTGGGCCTCTGGAAT | | GeneScript | Genomic-PCR | | |
| Tdgf1 reverse primer:  ATCCTGGGCTAATGGTGCAT | | GeneScript | Genomic-PCR | | |
| Stat3 forward primer:  TGCAGCTTACCTCCTCTTCC | | GeneScript | Genomic-PCR | | |
| Stat3 reverse primer:  GCAAGTTCAAGGACAGCTAAGG | | GeneScript | Genomic-PCR | | |
| Tet1 forward primer:  GAGTTGAACAAGTGGGGCTG | | GeneScript | Genomic-PCR | | |
| Tet1 reverse primer:  GGAGGAGGTACAGGTGATGG | | GeneScript | Genomic-PCR | | |
| Zfp42 forward primer:  GCCTTGCCTTGTTCCTGATT | | GeneScript | Genomic-PCR | | |
| Zfp42 reverse primer:  TCATTTGTTGACTACTGCCAAAG | | GeneScript | Genomic-PCR | | |
| Tet2 forward primer:  TCCAGGATCACACAGGAAGC | | GeneScript | Genomic-PCR | | |
| Tet2 reverse primer:  GTTGAGTCTCTGTGGCCTGT | | GeneScript | Genomic-PCR | | |
| Tet3 forward primer:  TTTTGTGTGCTTTCGTGGGT | | GeneScript | Genomic-PCR | | |
| Tet3 reverse primer:  TCAAATTGCCGGATGAGCTC | | GeneScript | Genomic-PCR | | |
| Tfcp2l1 forward primer:  TGAGGCTGGGGAGGTATGTA | | GeneScript | Genomic-PCR | | |
| Tfcp2l1 reverse primer:  CCATGCTAACCTGGGCTACA | | GeneScript | Genomic-PCR | | |
| Pecam1 forward primer:  TCTCTGTGTCGTATCCTGGC | | GeneScript | Genomic-PCR | | |
| Pecam1 reverse primer:  ACCAGCAGAGATTGAGCCAT | | GeneScript | Genomic-PCR | | |
| Klf5 forward primer:  GTCCACACACAAAAGCCCTT | | GeneScript | Genomic-PCR | | |
| Klf5 reverse primer:  AGTGAGTTCCAGGACAGCC | | GeneScript | Genomic-PCR | | |
| Sall4 forward primer:  TGGGGAATTGATTACTGCTGG | | GeneScript | Genomic-PCR | | |
| Sall4 reverse primer:  AAGCTGGCCTCGAACTCAG | | GeneScript | Genomic-PCR | | |
| Prdm14 forward primer:  TCTGCTGGCTTCTCATGGTC | | GeneScript | Genomic-PCR | | |
| Prdm14 reverse primer:  CCACATTGCCAAAATCACAGG | | GeneScript | Genomic-PCR | | |
| Klf15 forward primer:  ATGTGTGTCCCATCCCTTCC | | GeneScript | Genomic-PCR | | |
| Klf15 reverse primer:  AGCCCAGCCATCTATCCTTC | | GeneScript | Genomic-PCR | | |
| Klf4 forward primer:  TCTCTCTCCCTCCCTGTTCT | | GeneScript | Genomic-PCR | | |
| Klf4 reverse primer:  AAGAGATACACCCCAGCACC | | GeneScript | Genomic-PCR | | |
| Klf6 forward primer:  CTGTTAGGTTGGTGGGAGGG | | GeneScript | Genomic-PCR | | |
| Klf6 reverse primer:  AGTAAATGCCCACTAGCCCA | | GeneScript | Genomic-PCR | | |
| Klf17 forward primer:  GCTGTTCTAGATGGGACGCT | | GeneScript | Genomic-PCR | | |
| Klf17 reverse primer:  GACAGTGAAGGCCAGAGAGA | | GeneScript | Genomic-PCR | | |
| Pou3f1 forward primer:  GAGACCCAATAGCGAGCTCC | | GeneScript | Genomic-PCR | | |
| Pou3f1 reverse primer:  TGAGTTGGCGCATTCTGGAT | | GeneScript | Genomic-PCR | | |
| Lefty2 forward primer:  GTGGCCAACTGTGTCTTGTG | | GeneScript | Genomic-PCR | | |
| Lefty2 reverse primer:  GGACACACATGCGCAATAGT | | GeneScript | Genomic-PCR | | |
| Nr0b1 forward primer:  AAGCCAGGTCCCTCTTGTAC | | GeneScript | Genomic-PCR | | |
| Nr0b1 reverse primer:  GCATAATACACACCCCACCC | | GeneScript | Genomic-PCR | | |
| Lefty1 forward primer:  TGAACGGAACCCCTCACAAA | | GeneScript | Genomic-PCR | | |
| Lefty1 reverse primer:  GCCCACAATGCTTACCCATAG | | GeneScript | Genomic-PCR | | |
| Esrrb forward primer:  CCTGTAGTCCCAGTTTGTTCC | | GeneScript | Genomic-PCR | | |
| Esrrb reverse primer:  GGCCAGAAAGAATATCGCCC | | GeneScript | Genomic-PCR | | |
| Dppa3 forward primer:  CTCTGCCTCCCAAGTACAGG | | GeneScript | Genomic-PCR | | |
| Dppa3 reverse primer:  ATCACCCCAGTTTAAGGCCA | | GeneScript | Genomic-PCR | | |
| Klf2 forward primer:  CTATCTTGCCGTCCTTTGCC | | GeneScript | Genomic-PCR | | |
| Klf2 reverse primer:  ATGTGTCGCTTCATGTGCAA | | GeneScript | Genomic-PCR | | |
| Gbx2 forward primer:  GTGGGAGACGACGGACTG | | GeneScript | Genomic-PCR | | |
| Gbx2 reverse primer:  GAGGAGACAGTAGAGGCGAC | | GeneScript | Genomic-PCR | | |
| Foxd3 forward primer:  GAGGAAGGCTGGTGAAGTGA | | GeneScript | Genomic-PCR | | |
| Foxd3 reverse primer:  TGACACTTCGCCTTTTTGAA | | GeneScript | Genomic-PCR | | |
| Dppa2 forward primer:  TTGTCATTAGTCCCCACCCC | | GeneScript | Genomic-PCR | | |
| Dppa2 reverse primer:  AGTGTTGGGACTACAGGCTT | | GeneScript | Genomic-PCR | | |
| Gdf3 forward primer:  TGGAGGTATGAGGGAGGCTA | | GeneScript | Genomic-PCR | | |
| Gdf3 reverse primer:  ACATGGAGGAGACGCATGAT | | GeneScript | Genomic-PCR | | |
| Dnmt3a forward primer:  CTGAGTGATTGCCAGAGTGC | | GeneScript | Genomic-PCR | | |
| Dnmt3a reverse primer:  CAAAGTGGGTGTCAGAACGG | | GeneScript | Genomic-PCR | | |
| Dppa5a forward primer:  CGTGACCCGTAAAGATATCCC | | GeneScript | Genomic-PCR | | |
| Dppa5a reverse primer:  GTGGTAACCTTGGGATTCACA | | GeneScript | Genomic-PCR | | |
| Dnmt1 forward primer:  TGTGAGCGAAGGGAAGTCAT | | GeneScript | Genomic-PCR | | |
| Dnmt1 reverse primer:  CTGACCTCATCCCAGCCAA | | GeneScript | Genomic-PCR | | |
| Fbxo15 forward primer:  ACATGCAGAGAGAGCCAGTT | | GeneScript | Genomic-PCR | | |
| Fbxo15 reverse primer:  AATCCTCACCAACAAGCTGC | | GeneScript | Genomic-PCR | | |
| Dnmt3b forward primer:  AGCTGTGTAGTCTGTGCTGT | | GeneScript | Genomic-PCR | | |
| Dnmt3b reverse primer:  GTAGCAAAAGCATGGCCCTT | | GeneScript | Genomic-PCR | | |
| Dnmt3l forward primer:  ACACAGCCTGTAATCTCTGCA | | GeneScript | Genomic-PCR | | |
| Dnmt3l reverse primer:  CGTGGATGGAGCTCAGGAT | | GeneScript | Genomic-PCR | | |
| Sox1 forward primer:  CCTGTTTGCACAGTTCAGCC | | GeneScript | RT-PCR | | |
| Sox1 reverse primer:  CAGAGCCGGCAGTCATACAA | | GeneScript | RT-PCR | | |
| Sox2 forward primer:  TCTTCCTCCCACTCCAGG | | GeneScript | RT-PCR | | |
| Sox2 reverse primer:  GAAGCGCCTAACGTACCACT | | GeneScript | RT-PCR | | |
| Sox3 forward primer:  ACTGAACTCAAGAACCCCGT | | GeneScript | RT-PCR | | |
| Sox3 reverse primer:  TGCGAGTGCGAAGCGAT | | GeneScript | RT-PCR | | |
| Nanog forward primer:  TCAGAAATCCCTTCCCTCGC | | GeneScript | RT-PCR | | |
| Nanog reverse primer:  TGGTGGCTCACAACCATACG | | GeneScript | RT-PCR | | |
| Oct4 forward primer:  ATTGGGGAGGGAGAGGTGAA | | GeneScript | RT-PCR | | |
| Oct4 reverse primer:  TAGAGTGTGGTGAAGTGGGG | | GeneScript | RT-PCR | | |
| Fgf4 forward primer:  TTGCGTCCCTATTTGCTCTC | | GeneScript | RT-PCR | | |
| Fgf4 reverse primer:  GGTCCTTCTCACTGCCTCAG | | GeneScript | RT-PCR | | |
| Fgf5 forward primer:  GTGCACGGAGCAGTGAGAT | | GeneScript | RT-PCR | | |
| Fgf5 reverse primer:  GAAGTGGGTGGAGACGTGTT | | GeneScript | RT-PCR | | |
| Fgf8 forward primer:  AATCCAGCCCCAAACTACCC | | GeneScript | RT-PCR | | |
| Fgf8 reverse primer:  TGAAGGGCGGGTAGTTGAG | | GeneScript | RT-PCR | | |
| Fgfr1 forward primer:  CCAACCTCTAACCGCAGAAC | | GeneScript | RT-PCR | | |
| Fgfr1 reverse primer:  GCTTCAGCCACTGAATGTGA | | GeneScript | RT-PCR | | |
| Fgfr2 forward primer:  TCGCATTGGAGGCTATAAGG | | GeneScript | RT-PCR | | |
| Fgfr2 reverse primer:  ATCGATTCCCACTGCTTCAG | | GeneScript | RT-PCR | | |
| Fgfr3 forward primer:  GGTCTGGGCTAAGGATGGTA | | GeneScript | RT-PCR | | |
| Fgfr3 reverse primer:  TCTCAGCCACGCCTATGAAA | | GeneScript | RT-PCR | | |
| Fgfr4 forward primer:  GGAAGGAAATGTGGCTGCTC | | GeneScript | RT-PCR | | |
| Fgfr4 reverse primer:  TATAGTGACAGGCTGGCGAG | | GeneScript | RT-PCR | | |
| Inhba forward primer:  TAAACGAAGTTGCCCTTGCT | | GeneScript | RT-PCR | | |
| Inhba reverse primer:  GGTCCTGGTTCTGTTAGCCT | | GeneScript | RT-PCR | | |
| Acvr2a forward primer:  CTGCTGCAAAGTTGGCGTTC | | GeneScript | RT-PCR | | |
| Acvr2a reverse primer:  CTGGTGCCTCTTTTCTCTGC | | GeneScript | RT-PCR | | |
| Acvr2b forward primer:  CTTCTCTGGGGATCGCTGTG | | GeneScript | RT-PCR | | |
| Acvr2b reverse primer:  CATGCAGGTATGAGAGGCCT | | GeneScript | RT-PCR | | |
| Nodal forward primer:  CGCATCCTTCTTCTTCAAGC | | GeneScript | RT-PCR | | |
| Nodal reverse primer:  ACCCACACTCCTCCACAATC | | GeneScript | RT-PCR | | |
| Otx1 forward primer:  ACAGCTTGAAGGACTTCGCA | | GeneScript | RT-PCR | | |
| Otx1 reverse primer:  ATGGGGACCATGGAGGGTAT | | GeneScript | RT-PCR | | |
| Otx2 forward primer:  GCTCAGTCGCCACCTCTACT | | GeneScript | RT-PCR | | |
| Otx2 reverse primer:  ACCGGATCACCTCTGCTTTG | | GeneScript | RT-PCR | | |
| Otx3 forward primer:  AGCCCTTGGTAAAGTGGACG | | GeneScript | RT-PCR | | |
| Otx3 reverse primer:  GCCAGACAGTCAATTGGGGA | | GeneScript | RT-PCR | | |
| Grb2 forward primer:  TCAATGGGAAAGATGGCTTC | | GeneScript | RT-PCR | | |
| Grb2 reverse primer:  GACATAATTGCGGGGAAACA | | GeneScript | RT-PCR | | |
| Smc1b forward primer:  ATCAGAAGAGGCCTCAGTACA | | GeneScript | RT-qPCR | | |
| Smc1b reverse primer:  CAGCCAGTTCTGCCACTAAG | | GeneScript | RT-qPCR | | |
| Stra8 forward primer:  GGACCTCCTGACCATGAACA | | GeneScript | RT-qPCR | | |
| Stra8 reverse primer:  CTAGACAGGCCTCGGTGAG | | GeneScript | RT-qPCR | | |
| Dppa3 forward primer:  CTAACCCTAAACCCCGGTGT | | GeneScript | RT-qPCR | | |
| Dppa3 reverse primer:  TCAGCACCGACAACAAAGTG | | GeneScript | RT-qPCR | | |
| Hormad1 forward primer:  CCCCAGATTACCAACCACCA | | GeneScript | RT-qPCR | | |
| Hormad1 reverse primer:  TTTCCATTCGTTCCTTCTCAGTG | | GeneScript | RT-qPCR | | |
| D1Pas1 forward primer:  CCAGTTTCTTCAGTGACCGC | | GeneScript | RT-qPCR | | |
| D1Pas1 reverse primer:  CTCGTCTGCTTTGTCACACC | | GeneScript | RT-qPCR | | |
| Dazl forward primer:  GCAGCCACGTCCTTTGATTT | | GeneScript | RT-qPCR | | |
| Dazl reverse primer:  CCTGAACTGGTGAACTTGGA | | GeneScript | RT-qPCR | | |
| Nr0b1 forward primer:  GCTGCTTTTGTGGGGAGAAT | | GeneScript | RT-qPCR | | |
| Nr0b1 reverse primer:  TATAAGATGCTGCCCTGCCT | | GeneScript | RT-qPCR | | |
| Tktl2 forward primer:  GGACTTGCTGAACTTGCGAA | | GeneScript | RT-qPCR | | |
| Tktl2 reverse primer:  GGTAGCTGGCCTTGTCAAAG | | GeneScript | RT-qPCR | | |
| Slc25a31 forward primer:  CCATCGAGCGTGTGAAGC | | GeneScript | RT-qPCR | | |
| Slc25a31 reverse primer:  ACATTTGCCAAATTGCCACG | | GeneScript | RT-qPCR | | |
| Trim52 forward primer:  GCAAGGAAGATGAGCAGGAC | | GeneScript | RT-qPCR | | |
| Trim52 reverse primer:  TTCCTCGTCATCCCACTCAG | | GeneScript | RT-qPCR | | |
| Rpl10l forward primer:  AAGAAGGCGAAGGTGGATGA | | GeneScript | RT-qPCR | | |
| Rpl10l reverse primer:  GATGCGGATGACATGGAAGG | | GeneScript | RT-qPCR | | |
| Pecam1 forward primer:  AGGACGATGCGATGGTGTAT | | GeneScript | RT-qPCR | | |
| Pecam1 reverse primer:  ATGCACCTTCACCTCGTACT | | GeneScript | RT-qPCR | | |
| Gpat2 forward primer:  AGCATAATGGACCTGGGCTT | | GeneScript | RT-qPCR | | |
| Gpat2 reverse primer:  CCATGATCTTCTGGGAGGCA | | GeneScript | RT-qPCR | | |
| Sycp1 forward primer:  AATTGAAGCCCAGCGAAAAG | | GeneScript | RT-qPCR | | |
| Sycp1 reverse primer:  GCAGATCTAGCACAGGTTTCC | | GeneScript | RT-qPCR | | |
| Tuba3a forward primer:  TGTGTCGTCCATTACAGCCT | | GeneScript | RT-qPCR | | |
| Tuba3a reverse primer:  GACAGCTGCTCATGGTATGC | | GeneScript | RT-qPCR | | |
| Fbxo15 forward primer:  ATTTGGGTCAGGAAGTACGC | | GeneScript | RT-qPCR | | |
| Fbxo15 reverse primer:  TTGACAGGACTGAGGCTGTT | | GeneScript | RT-qPCR | | |
| Tbx3 forward primer:  CATCCCTTCCTACCTCACCG | | GeneScript | RT-qPCR | | |
| Tbx3 reverse primer:  AGGGATGAGAAAGGGATGCC | | GeneScript | RT-qPCR | | |
| Piwil2 forward primer:  CTCCAGCTCTGTCTCCAACT | | GeneScript | RT-qPCR | | |
| Piwil2 reverse primer:  ACTGCTTCGTTATGACACTGG | | GeneScript | RT-qPCR | | |
| Rbm44 forward primer:  TAGAGAGTGGTCCTAGCCCC | | GeneScript | RT-qPCR | | |
| Rbm44 reverse primer:  TGGTTTAAGGGAGAGCTGCT | | GeneScript | RT-qPCR | | |
| Mael forward primer:  CCTCCTCATTGTGAACAGCG | | GeneScript | RT-qPCR | | |
| Mael reverse primer:  ACACAGTTGCTTGGTCATGC | | GeneScript | RT-qPCR | | |
| Gbx2 forward primer:  CTACCCCATGTTCATGCCCT | | GeneScript | RT-qPCR | | |
| Gbx2 reverse primer:  ATGAGCGTGGAGGTGAGC | | GeneScript | RT-qPCR | | |
| Zfp42 forward primer:  AAGGGGACGAAGCAAGAGAA | | GeneScript | RT-qPCR | | |
| Zfp42 reverse primer:  TCCCTCAGCTTCTTCTTGCA | | GeneScript | RT-qPCR | | |
| Klf4 forward primer:  GACATCAATGACGTGAGCCC | | GeneScript | RT-qPCR | | |
| Klf4 reverse primer:  TGGGCTTCCTTTGCTAACAC | | GeneScript | RT-qPCR | | |
| Esrrb forward primer:  TAGTTGCGGCTCCTTCATCA | | GeneScript | RT-qPCR | | |
| Esrrb reverse primer:  GTACAGTCCTCGTAGCTCTTG | | GeneScript | RT-qPCR | | |
| Nanog forward primer:  ATGCGGACTGTGTTCTCTCA | | GeneScript | RT-qPCR | | |
| Nanog reverse primer:  CCGCTTGCACTTCATCCTTT | | GeneScript | RT-qPCR | | |
| Klf2 forward primer:  GTTGCACTACGGGCCTCC | | GeneScript | RT-qPCR | | |
| Klf2 reverse primer:  CTGCAAGTATGTGTGGCGG | | GeneScript | RT-qPCR | | |
| Gdf3 forward primer:  CTGACTCTAGACTTGGGGCC | | GeneScript | RT-qPCR | | |
| Gdf3 reverse primer:  CTCCAATCCTTAAGCGCACC | | GeneScript | RT-qPCR | | |
| Cdh1 forward primer:  GGACAGCCTTCTTTTCGGAA | | GeneScript | RT-qPCR | | |
| Cdh1 reverse primer:  TCACTTTGGTAGACAGCTCCC | | GeneScript | RT-qPCR | | |
| Pou5f1 forward primer:  GGATGGCATACTGTGGACCT | | GeneScript | RT-qPCR | | |
| Pou5f1 reverse primer:  TCTCCAACTTCACGGCATTG | | GeneScript | RT-qPCR | | |
| Acvr2b forward primer:  CTGTGCGGACTCCTTTAAGC | | GeneScript | RT-qPCR | | |
| Acvr2b reverse primer:  CACAGCCACAAAGTCGTTCA | | GeneScript | RT-qPCR | | |
| Tdgf1 forward primer:  TTTCCAGTTTGTGCCTTCCG | | GeneScript | RT-qPCR | | |
| Tdgf1 reverse primer:  GCACAGGGAACACTTCTTGG | | GeneScript | RT-qPCR | | |
| Myc forward primer:  AAATTCGAGCTGCTTCCCAC | | GeneScript | RT-qPCR | | |
| Myc reverse primer:  CTCGGTCATCATCTCCAGCT | | GeneScript | RT-qPCR | | |
| Nodal forward primer:  CACTGGAAAAGCAGGTGTCC | | GeneScript | RT-qPCR | | |
| Nodal reverse primer:  GGCTTCTGTCTGGCAAATGA | | GeneScript | RT-qPCR | | |
| Cldn6 forward primer:  ACTCCAAGTCTCGTCTGGTG | | GeneScript | RT-qPCR | | |
| Cldn6 reverse primer:  CATAGTAGCCCTCCACCCAG | | GeneScript | RT-qPCR | | |
| Pitx2 forward primer:  GTCTCTGTCCACCAAGAGCT | | GeneScript | RT-qPCR | | |
| Pitx2 reverse primer:  GCTGCTCAGGTTGTTCAAGT | | GeneScript | RT-qPCR | | |
| Gata6 forward primer:  CCCACTTCTGTGTTCCCAATTG | | GeneScript | RT-qPCR | | |
| Gata6 reverse primer:  TTGGTCACGTGGTACAGGCG | | GeneScript | RT-qPCR | | |
| Unc5b forward primer:  TCCCAGACTCCTACCCATCA | | GeneScript | RT-qPCR | | |
| Unc5b reverse primer:  CCCACTCGCCATTACACTTG | | GeneScript | RT-qPCR | | |
| Car4 forward primer:  CCCAGACTGACACCCTTCAT | | GeneScript | RT-qPCR | | |
| Car4 reverse primer:  TGCAACTGTACAGCCTCGTA | | GeneScript | RT-qPCR | | |
| Sema6a forward primer:  TCGCATTAATGGCCGTGATG | | GeneScript | RT-qPCR | | |
| Sema6a reverse primer:  GGTGTCCAGGTAGAGTCAGG | | GeneScript | RT-qPCR | | |
| Eomes forward primer:  TGCTCGCTCTTCCAGTACC | | GeneScript | RT-qPCR | | |
| Eomes reverse primer:  GTAGAGCGGAGAACCCTGG | | GeneScript | RT-qPCR | | |
| Pim2 forward primer:  ATATCACAGAGAAGGGGCCG | | GeneScript | RT-qPCR | | |
| Pim2 reverse primer:  AGTCAGTGTACGGCTCATCG | | GeneScript | RT-qPCR | | |
| Sox17 forward primer:  GCCGAGCCAAAGCGG | | GeneScript | RT-qPCR | | |
| Sox17 reverse primer:  GTCAACGCCTTCCAAGACTTG | | GeneScript | RT-qPCR | | |
| Sox3 forward primer:  CAACTCCGAGATCAGCAAGC | | GeneScript | RT-qPCR | | |
| Sox3 reverse primer:  CTTCTTGAGCAGCGTCTTGG | | GeneScript | RT-qPCR | | |
| Vrtn forward primer:  GCAACATCAAGATCCGACCC | | GeneScript | RT-qPCR | | |
| Vrtn reverse primer:  GTAGCAGAGGACCCAGGTTT | | GeneScript | RT-qPCR | | |
| Otx2 forward primer:  GCTATGCTGGCTCAACTTCC | | GeneScript | RT-qPCR | | |
| Otx2 reverse primer:  GGAAAGAGAAGCTGGGGACT | | GeneScript | RT-qPCR | | |
| Acta2 forward primer:  GGGATCCTGACGCTGAAGTA | | GeneScript | RT-qPCR | | |
| Acta2 reverse primer:  CATACATGGCGGGGACATTG | | GeneScript | RT-qPCR | | |
| Eno1b forward primer:  GCCCTAGAACTCCGAGACAA | | GeneScript | RT-qPCR | | |
| Eno1b reverse primer:  ATTCTCTGTGCCGTCCATCT | | GeneScript | RT-qPCR | | |
| Car2 forward primer:  ATAAAGCTGCGTCCAAGAGC | | GeneScript | RT-qPCR | | |
| Car2 reverse primer:  CATCAGATGAGCCCCAGTGA | | GeneScript | RT-qPCR | | |
| Psors1c2 forward primer:  GACCCTTCTCCCGGATCTAC | | GeneScript | RT-qPCR | | |
| Psors1c2 reverse primer:  GAAGATCTCTCCAGGGACGG | | GeneScript | RT-qPCR | | |
| Fgf8 forward primer:  GGAACCCAGCTGACACTCTC | | GeneScript | RT-qPCR | | |
| Fgf8 reverse primer:  GCTGTGTGACTTTAGGCAGG | | GeneScript | RT-qPCR | | |
| Gal forward primer:  TGCCTGCAAAGGAGAAGAGA | | GeneScript | RT-qPCR | | |
| Gal reverse primer:  CGGACAATGTTGCTCTCAGG | | GeneScript | RT-qPCR | | |
| Psmb8 forward primer:  AGTACTGGGAGAGGCTGTTG | | GeneScript | RT-qPCR | | |
| Psmb8 reverse primer:  TTGTCCCAGCCACAGATCAT | | GeneScript | RT-qPCR | | |
| Enpp2 forward primer:  TCGGCGTCAATCTCTGCTTA | | GeneScript | RT-qPCR | | |
| Enpp2 reverse primer:  TCACACCGACAGTCAGGAG | | GeneScript | RT-qPCR | | |
| T forward primer:  TCCCGGTGCTGAAGGTAAAT | | GeneScript | RT-qPCR | | |
| T reverse primer:  CAAAATTGGGCGAGTCTGGG | | GeneScript | RT-qPCR | | |
| Fgf5 forward primer:  TTGCGACCCAGGAGCTTAAT | | GeneScript | RT-qPCR | | |
| Fgf5 reverse primer:  CTACGCCTCTTTATTGCAGC | | GeneScript | RT-qPCR | | |
| Foxa2 forward primer:  CCCTACGCCAACATGAACTCG | | GeneScript | RT-qPCR | | |
| Foxa2 reverse primer:  GTTCTGCCGGTAGAAAGGGA | | GeneScript | RT-qPCR | | |
| Cer1 forward primer:  GCTTGTTCTCTTGCCTCTGG | | GeneScript | RT-qPCR | | |
| Cer1 reverse primer:  TGGACAGCATCTTCCCTCTC | | GeneScript | RT-qPCR | | |
| Dkk1 forward primer:  TGGCCGTGTTTACAATGATG | | GeneScript | RT-qPCR | | |
| Dkk1 reverse primer:  TACTTGTTCCCGCCCTCATA | | GeneScript | RT-qPCR | | |
